# Extended Reality Interventions for Health and Procedural Anxiety: Panoramic Meta-Analysis Based on Overviews of Reviews

**DOI:** 10.2196/58086

**Published:** 2025-01-08

**Authors:** Tom Arthur, GJ Melendez-Torres, David Harris, Sophie Robinson, Mark Wilson, Sam Vine

**Affiliations:** 1 Faculty of Health and Life Sciences University of Exeter Exeter United Kingdom

**Keywords:** virtual reality, exposure therapy, distraction techniques, patient education, fear, phobia, immersive, anxiety, meta-analysis, extended reality, intervention, procedural anxiety, patient anxiety, exposure, distraction

## Abstract

**Background:**

Extended reality (XR) technologies are increasingly being used to reduce health and procedural anxieties. The global effectiveness of these interventions is uncertain, and there is a lack of understanding of how patient outcomes might vary between different contexts and modalities.

**Objective:**

This research used panoramic meta-analysis to synthesize evidence across the diverse clinical contexts in which XR is used to address common outcomes of health and procedural anxiety.

**Methods:**

Review-level evidence was obtained from 4 databases (MEDLINE, Embase, APA PsycINFO, and Epistemonikos) from the beginning of 2013 until May 30, 2023. Reviews that performed meta-analysis of randomized controlled trials relating to patient-directed XR interventions for health and procedural anxiety were included. Studies that analyzed physiological measures, or focused on technologies that did not include meaningful immersive components, were excluded. Furthermore, data were only included from studies that compared intervention outcomes against no-treatment or treatment-as-usual controls. Analyses followed a preregistered, publicly available protocol. Trial effect sizes were extracted from reviews and expressed as standardized mean differences, which were entered into a 3-level generalized linear model. Here, outcomes were estimated for patients (level 1), studies (level 2), and anxiety indications (level 3), while meta-regressions explored possible influences of age, immersion, and different mechanisms of action. Where relevant, the quality of reviews was appraised using the AMSTAR-2 (A Measurement Tool to Assess Systematic Reviews, Revised Instrument) tool.

**Results:**

Data from 83 individual trials were extracted from 18 eligible meta-analyses. Most studies involved pediatric patient groups and focused on procedural, as opposed to general, health anxieties (eg, relating to needle insertion, dental operations, and acute surgery contexts). Interventions targeted distraction-, education-, and exposure-based mechanisms, and were provided via a range of immersive and nonimmersive systems. These interventions proved broadly effective in reducing patient anxiety, with models revealing significant but heterogeneous effects for both procedural (*d*=–0.75, 95% CI –0.95 to –0.54) and general health (*d*=–0.82, 95% CI –1.20 to –0.45) indications (when compared with nontreatment or usual-care control conditions). For procedural anxieties, effects may be influenced by publication bias and appear more pronounced for children (vs adults) and nonimmersive (vs immersive) technology interventions, but they were not different by indication.

**Conclusions:**

Results demonstrate that XR interventions have successfully reduced patient anxiety across diverse clinical contexts. However, significant uncertainty remains about the generalizability of effects within various unexplored indications, and existing evidence is limited in methodological quality. Although current research is broadly positive in this area, it is premature to assert that XR interventions are effective for any given health or procedural anxiety indication.

## Introduction

Health and procedural anxiety can lead to delayed engagement with treatment, reduced treatment adherence, worsening symptoms, and compromised quality of life. Two primary approaches to alleviate health and procedural anxiety are exposure and distraction therapies. Distraction-based approaches seek to reduce anxiety by diverting attention away from distressing thoughts, situations, or physical sensations. While controversial as a long-term technique [1], these are effective for short-term anxiety reduction [2]. However, distraction methods may not always be readily available or accessible, particularly in moments of acute anxiety/stress. Exposure therapy has been traditionally delivered in vivo. By directly facing the fearful stimuli without resorting to escape, avoidance, or rituals, the patient’s connection between the stimulus and memory structures is modified, leading to a potential reduction in the elicited anxiety response [3]. While effective, in vivo exposure has several limitations; it can be difficult to instigate when the anxiety-inducing environment or stimulus is inaccessible and can be time-consuming and expensive to simulate. There is, therefore, a drive toward the use of technologies that can deliver these therapies in a more cost-effective and accessible manner.

Extended reality (XR) technologies—including virtual reality, augmented reality, and mixed reality—are well suited to deliver these therapies [4]. XR immerses users in a digital environment, providing a heightened sense of presence and interaction with synthetic sensory stimuli. These simulations can feel realistic, enabling users to practice skills or experience environments in a controlled and immersive setting that can ultimately aid the therapeutic process. For example, XR can gradually expose patients to anxiety-inducing cues, to elicit realistic emotional responses that can become habituated over time, and they can also supply experiential patient education material about a procedure or condition. Similarly, the highly immersive and interactive nature of XR makes it an ideal distraction tool, which can divert attention toward more pleasurable or relaxing sensory environments. From a practical perspective, XR interventions can be used to address wide-ranging anxiety indications, given the breadth of stimuli that can now be simulated effectively and the capability to be delivered remotely (eg, headsets could be posted to patient’s homes before attending appointments). They are also considered highly engaging forms of therapy [5] that can provide personalized patient experiences (eg, by tailoring exposure- and distraction-based cues to individual user data), so they may promote better treatment adherence [6]. XR is therefore a scalable tool that has the potential to enhance the accessibility of therapeutic interventions for patients who may be unwilling or unable to participate in more conventional forms of psychological treatment [7].

Panoramic meta-analyses are a tool to integrate evidence from trials covering the same intervention and the same outcomes over a range of indications. Originally designed for use in the context of overviews of reviews (ie, systematic reviews of systematic reviews), early panoramic meta-analyses addressed topics such as sutures versus staples for wound healing in a range of surgical sites [8]. More recently, panoramic meta-analyses have also addressed mental health outcomes, such as cognitive behavioral therapy for health-related quality of life [9]. When used to evaluate evidence for a given intervention method, panoramic meta-analyses offer a statistical advantage for random effects modeling, in that they generate more stable estimates of between-study variance. But more importantly, they generate “global” estimates of effectiveness that allow for (1) assessment of likely future effects via prediction intervals; (2) assessment of the relative importance of indication in explaining effect heterogeneity via variance partitioning; and (3) tests of effect modification (ie, via meta-regression) that may be better powered by drawing on evidence from multiple indications. Thus, in this research, panoramic meta-analyses can indicate the broad impact that XR interventions have on patient anxiety across diverse clinical contexts, while also offering unique insight into the exchangeability of effects between different intervention types, technologies, populations, and indications. From an applied perspective, one can use this insight to inform future clinical programs (eg, by deciding whether to adopt XR based on pooled data that reflects efficacy or inefficacy over general/specific contexts, or by using findings that indicate whether certain indications/methods are associated with greater efficacy or uncertainty).

Consequently, a panoramic meta-analysis was undertaken to synthesize evidence across the diverse health-related contexts in which XR is used to address common outcomes of health and procedural anxiety. The aim of this analysis was to consider the global effectiveness of XR, to assess the likelihood of effectiveness for other clinical indications, and to explore whether key intervention characteristics are associated with varying levels of effectiveness.

## Methods

### Overview

A protocol for this review was registered on the Open Science Framework [10], a widely used, publicly available platform that permits the open sharing of research data and material. This analysis is part of a larger evidence-synthesis project focusing on XR interventions for health and procedural anxiety.

### Included Studies

A set of systematic reviews was identified, which included meta-analyses of randomized controlled trials relating to patient-directed XR interventions for health and procedural anxiety outcomes published from 2013 onward. In line with the panoramic meta-analysis approach, these reviews informed the sample of trials and effect sizes that were subsequently examined in generalized linear models (see the Statistical Methods section below). Specific inclusion and exclusion criteria are detailed in Table 1. Procedural anxiety was defined as anxiety both related and proximal to specific medical procedures, generally as a measure of state anxiety [11]. Health anxiety was defined as anxiety relating to broader health conditions, generally as a measure of trait anxiety [12]. Studies that based their analyses on physiological measures of anxiety were excluded (eg, blood pressure and heart rate), and data were only extracted that compared intervention outcomes against no-treatment or treatment-as-usual controls. Eligibility criteria did not impose any further stipulations for the trial-level data, and the extraction of effect sizes was not restricted to studies from any given language, date, or region.

**Table 1:** Eligibility criteria.

Characteristics	Inclusion criteria	Exclusion criteria
Study design	Systematic reviews^a^ of randomized controlled trials that included meta-analyses or forest plots with reported effect sizes Reviews published as full-text articles in peer-reviewed journals from 2013 onward^b^ Randomized controlled trials that feature in these systematic reviews	Reviews that did not perform adequate searches of an electronic database, using the structured search query, with inclusion/exclusion criteria specified Conference abstracts, dissertations, and theses Nonrandomized trials
Interventions	Focus on patient-directed XR^c^ interventions^d^ in any primary or discrete subgroup analyses	Focus on technologies that do not include meaningful immersive components (eg, mobile apps)
Comparators	Data comparisons against no-treatment or treatment-as-usual controls	None
Outcome	Patient health or procedural anxiety measures	Trial data relating to pain outcomes or physiological indicators of anxiety

^a^Definitions of systematic reviews were based on criteria developed for the Database of Abstracts of Reviews of Effects.

^b^Trial eligibility was not restricted to studies from any given date.

^c^XR: extended reality.

^d^XR interventions included virtual reality, mixed reality, or augmented reality systems.

### Search and Selection

Searches were carried out on May 30, 2023, in MEDLINE and Embase (on the Ovid platform), APA PsycINFO (in Ovid), and Epistemonikos. This variety of databases was selected to cover general medical literature, specialist psychology sources, and a collection of systematic reviews. Searches used appropriate subject headings and keywords for the intervention (virtual reality), outcome (health anxiety), and study design (systematic reviews), with full terms and methods specified in Multimedia Appendix 1. Records were screened independently and in duplicate, both at the title and abstract stage and at full-text stage, recording reasons for exclusion at full-text and involving a third reviewer in case of disagreement.

### Data Extraction, Risk of Bias, and Classification

Informed by included reviews and relevant trials within them, a set of high-level indications were inductively generated as distinct “use cases” of XR. Different classifications were developed for procedural anxiety and general health anxiety. Within each indication and outcome, the highest quality, the most recent review was prioritized for effect size extraction from included forest plots, defining quality using the results of appraisal with the AMSTAR-2 (A Measurement Tool to Assess Systematic Reviews, Revised Instrument) [13]. The analysis then proceeded through the remaining reviews sequentially, organizing reviews by publication year and, within year, by quality, until no additional trials were identified, extracting evidence from randomized controlled trials only and preferring effect estimates for the same trial and outcome from higher-priority reviews. Where available, trial-level risk of bias (RoB) assessments were extracted, from included reviews providing sufficient evidence. Extraction items are provided in the protocol. The original trial was consulted when (1) extracted effect sizes were inappropriate or possibly inaccurate (eg, a standardized mean difference greater than 2) or (2) effect sizes were not presented as standardized mean differences and relevant standard deviations were not presented in included plots. One reviewer led extraction with auditing by a second.

### Statistical Methods

All effect estimates were expressed as standardized mean differences, where negative values are positive (ie, greater reduction in anxiety). For each outcome, a 1-step meta-analysis model was estimated using a 3-level generalized linear model, where level 1 (patients within trials) is implied, level 2 is the study-level effect estimate, and level 3 is the indication. All models were estimated using restricted maximum likelihood and random effects at level 2 and level 3, with a common level 2 variance component across all indications to stabilize estimation. Heterogeneity was quantified using a standard variance partitioning method, generating a between-study, within-indication *I*^2^, and a between-indication *I*^2^ based on the Higgins and Thompson method. Statistical analyses were undertaken on Stata (version 18; StataCorp).

### Sensitivity and Exploratory Analyses

The registered protocol stated that for any instances containing too few indications in an outcome (fewer than 10), sensitivity analysis would be undertaken with a 2-level model, using fixed effects for indication at level 2. This model was compared with an unconditional meta-analysis model using a likelihood ratio test as an additional assessment of the value of indication as a grouping variable. Additionally, protocol-specified meta-regressions by age group (adult vs children), system immersion (immersive vs nonimmersive), and mechanism of action (relaxation or distraction vs exposure or education) were undertaken, where sufficient variation was present. Finally, funnel plots were drawn as an exploratory analysis, and an Egger test was estimated to examine small-study bias where the number of trials for any one indication and outcome was greater than 10.

## Results

### Search Results

**Figure 1:**
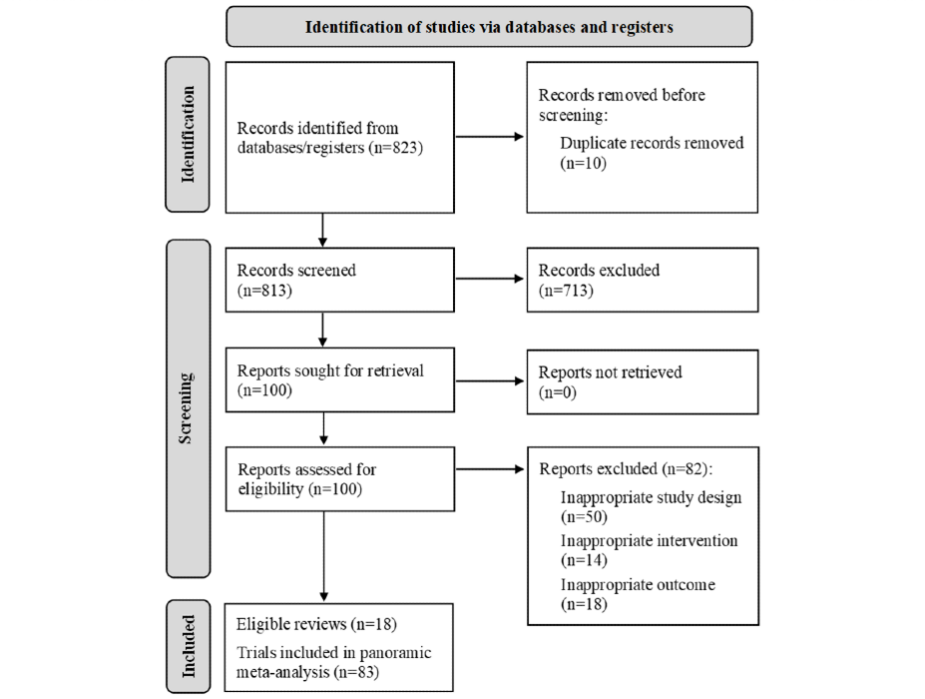
PRISMA (Preferred Reporting Items for Systematic Reviews and Meta-Analyses) flowchart of retrieved, screened, and included studies.

A total of 18 meta-analyses informed the sample of trials, of which 10 provided procedural anxiety outcomes and 8 provided general health anxiety outcomes. Across these meta-analyses, 53 trial effects for procedural health anxiety outcomes and 30 trial effects for general health anxiety outcomes were examined. Two studies [14,15] reported data from multiple XR interventions, which were examined separately. Analyses excluded 15 trials that other meta-analyses included: 14 did not meet the specified definitions of XR, and one used physiological measures only. Study selection processes are outlined in Figure 1, via a PRISMA (Preferred Reporting Items for Systematic Reviews and Meta-Analyses) flowchart (see full list of included reviews in Multimedia Appendix 2 [16-33]).

### Trial Characteristics

Details of study-level extraction, measures used, and interventions are provided in Multimedia Appendix 3. Reviews examined anxiety indications relating to either a specific medical procedure (n=8) or those additionally relating to general health conditions and prolonged treatment pathways (n=10). The average number of trials examined within each review was 6.67 (SD 3.38), although 18 overlapping trials were detected in multiple meta-analyses. Trial-specific RoB assessments or quality appraisals were reported in all but 3 of the included studies and are presented in Multimedia Appendix 4. These methodological evaluations followed inconsistent assessment criteria (eg, the Cochrane RoB, ROBINS-I, and Delphi List tools); however, only 8 trials were considered to have low RoB overall and 49 were deemed to have a high risk or some concerns. Methods for assessing anxiety notably varied between trials and were dependent on clinical indications. The most common measures were the Modified Dental Anxiety Scale (for dental studies), the State-Trait Anxiety Inventory (for cancer treatment, cardiac rehabilitation), the Modified Yale Preoperative Anxiety Scale (for nondental surgical operations), the Self-rating Anxiety Scale (for maternity), the Observational Scale of Behavioral Distress for radiographic procedures (for imaging), the Pain Anxiety Symptoms Scale (for chronic pain conditions), and simple visual analog scales (for wound care, needle-related anxieties).

Most of the included trial analyses (44/83, 53%) evaluated anxiety in pediatric patients only, while 36 trials focused on adults only, and 3 trials involved mixed-age samples (ie, children and adults). From an intervention perspective, the vast majority of procedural anxiety interventions (46/53, 55%) included just a single XR-based therapy session, the duration of which varied from 1 minute [34] to 90 minutes [35] (based on the context and population). Interventions for general health anxieties were more heterogeneous in design, ranging from single sessions to repeated activities over a period of months (Multimedia Appendix 3). Immersive forms of XR were adopted in 37 study interventions, whereas 46 interventions used nonimmersive methods. Interventions incorporated a range of XR technologies, including standalone virtual reality systems, portable glasses, mobile phone head-mounted displays, and monitor-based simulation devices.

In total, 66 interventions were classed as distraction-based or relaxation-based. These included natural scenery experiences (28 trial interventions), adventure or rollercoaster games (22 trial interventions), cartoons (12 trial interventions), mindfulness applications (1 trial intervention), and 3D images/videos (2 trial interventions). Conversely, 16 interventions were classified as education- or exposure-based. Separations between education- and exposure-based methods were not made, as numerous interventions incorporated elements of both components, and studies rarely provided sufficient details or rationale about XR procedures. This was also the case for distraction- and relaxation-based approaches (although it is also worth noting that all distraction experiences could be considered somewhat relaxing relative to anxiety-inducing medical stimuli).

### Procedural Anxiety

#### Overview of Trials

**Figure 2:**
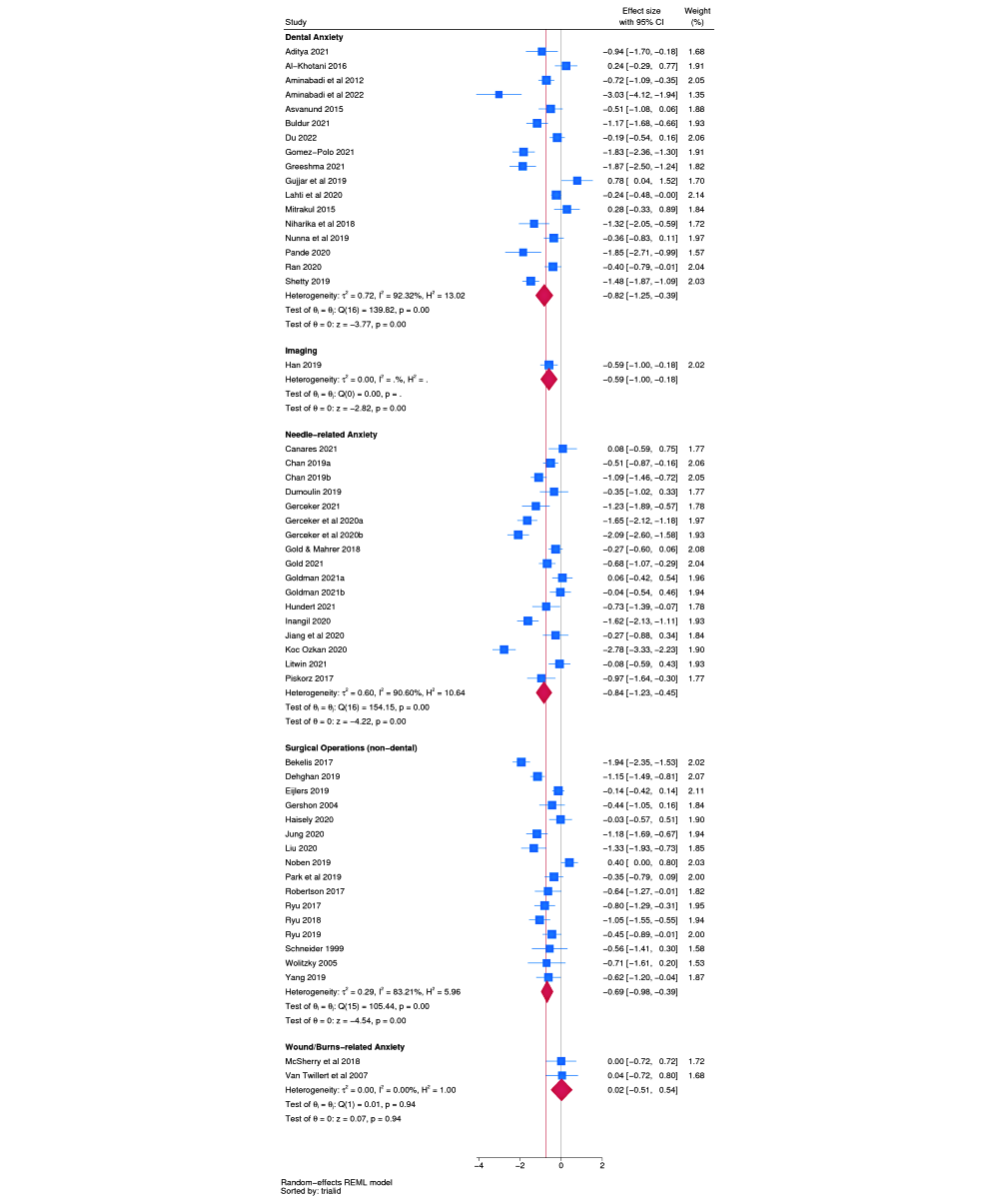
Forest plot for procedural anxiety outcomes.

Classification of trials measuring procedural anxiety outcomes identified 5 indications: dental anxiety (n=17), imaging (n=1), needle-related anxiety (n=17), nondental surgical operations (n=16), and anxiety related to wounds and burn care (n=2). Indication-specific meta-analyses are presented in Figure 2. In brief, all interventions generated significant effects within indication, except for interventions for wounds and burn care.

#### Panoramic Meta-Analysis

A panoramic meta-analysis model nesting trials within indication generated an overall pooled effect (*d*) of –0.75 (95% CI –0.95 to –0.54) (Table 2). The between-indication (level 3) variance parameter for this model was equal to 0, whereas the within-indication, between-trial (level 2) variance parameter was equal to 0.48. Thus, the *I*^2^ attributable to indication was 0%, and the *I*^2^ attributable to between-trial differences was 89.0%. However, a prediction interval for the pooled effect spanned –2.11 to 0.62, indicating that effectiveness in a future trial would be highly uncertain.

**Table 2:** Panoramic meta-analysis models.

Model					SMD^a^ (95% CI)			Between-indication τ^2^			Between-trial τ^2^
**Procedural anxiety**											
	**Overall**			–0.75 (–0.95 to –0.55)			0.00			0.48	
	**Age**						0.00			0.45	
		Adults	–0.27 (–0.73 to 0.18)								
		Children	–0.86 (–1.07 to –0.64)								
		Difference	–0.58 (–1.08 to –0.08)								
	**Immersive**						0.00			0.44	
		Nonimmersive	–0.97 (–1.22 to –0.71)								
		Immersive	–0.46 (–0.75 to –0.16)								
		Difference	0.51 (0.12 to 0.90)								
	**Mechanism**						0.00			0.48	
		Relaxation/distraction	–0.82 (–1.05 to –0.58)								
		Education/exposure	–0.53 (–0.95 to –0.11)								
		Difference	0.28 (–0.19 to 0.76)								
**General health anxiety**											
	**Overall**			–0.82 (–1.29 to –0.45)			0.09			0.33	
	**Immersive**						0.09			0.35	
		Nonimmersive	–0.86 (–1.30 to –0.41)								
		Immersive	–0.78 (–1.25 to –0.30)								
		Difference	0.08 (–0.44 to 0.60)								

^a^SMD: standardized mean difference.

#### Sensitivity Analyses

Because the number of indications was fewer than 10, a protocol-specified sensitivity analysis was undertaken, treating indication as a fixed effect. A likelihood ratio test comparing this model against a simple 2-level model suggested that including an indication as a fixed effect did not improve model fit (*χ*^2^_4_=2.47, *P*=.65).

Three protocol-specified meta-regressions were then undertaken. Because of the size of each indication category, these should be regarded as indicative only. Across indications, interventions were significantly more effective in children versus adults (difference: *d*=–0.58, 95% CI –1.08 to –0.08), and immersive interventions appeared less effective than nonimmersive interventions (difference: *d*=0.51, 95% CI 0.12-0.90). There was no significant difference in the size of effect between interventions using relaxation or distraction and interventions using education or exposure.

An Egger test for the dental anxiety indication implied some evidence of small-study bias (*P*=.048), with a funnel plot reflecting this possibility (Multimedia Appendix 5). While an Egger test for needle-related anxiety did not generate a significant result (*P*=.97), a funnel plot indicated significant unexplained heterogeneity. An Egger test for surgical operations was not significant (*P*=.86), and a funnel plot did not suggest evidence of small-study bias.

### General Health Anxiety

#### Overview of Trials

**Figure 3:**
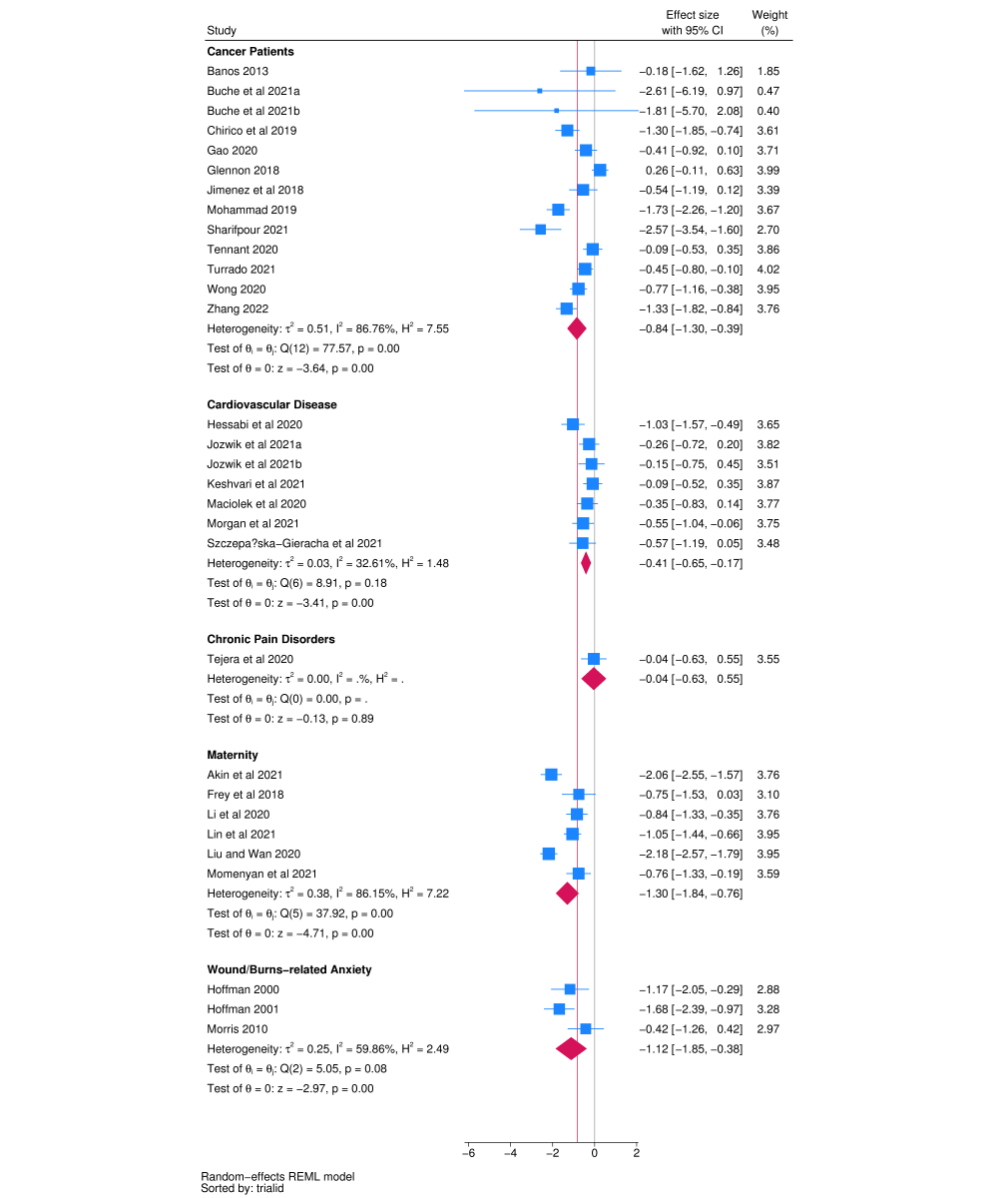
Forest plot for general health anxiety outcomes.

Classification of trials measuring general health anxiety outcomes identified 5 indications: cancer (n=13), cardiovascular disease (n=7), chronic pain disorders (n=1), maternity (n=6), and anxiety related to wounds and burn care (n=3). Indication-specific meta-analyses are presented in Figure 3. All interventions generated significant effects within the indication, except for interventions for chronic pain disorders.

#### Panoramic Meta-Analysis

A panoramic meta-analysis model nesting trials within indication generated an overall pooled effect (*d*) of –0.82 (95% CI –1.20 to –0.45; Table 2). The between-indication (level 3) variance parameter for this model was equal to 0.09, whereas the within-indication, between-trial (level 2) variance parameter was equal to 0.33. Thus, the *I*^2^ attributable to indication was 17.7%, and the *I*^2^ attributable to between-trial differences was 67.3%. The conditional *I*^2^ using only trial-level and between-trial variance was 81.8%. The prediction interval for the pooled effect spanned –2.09 to 0.45, indicating, as for procedural anxiety, the highly uncertain effectiveness of XR interventions in any future trial.

#### Sensitivity Analyses

Since the number of indications was fewer than 10, a protocol-specified sensitivity analysis was undertaken, which treated indication as a fixed effect. A likelihood ratio test comparing this model against a simple 2-level model suggested that including indication as a fixed effect did not improve model fit (χ^2^_4_=8.57, *P*=.07), albeit with a marginal significance test.

Of the 3 protocol-specified meta-regression analyses, tests for age and mechanism were not undertaken, as only 2 indications had a variation on these meta-regressors. The difference between immersive and nonimmersive interventions was small and not significant (difference: *d*=0.08; 95% CI –0.44 to 0.60). Finally, an Egger test for trials in the cancer indication generated a nonsignificant result (*P*=.19), and a funnel plot did not suggest conclusive evidence of small-study bias (Multimedia Appendix 5).

## Discussion

### Principal Findings

Using an innovative statistical method, this analysis demonstrated that the effectiveness of XR interventions for both procedural and health anxiety is heterogeneous but conclusive; however, the evidence base for procedural anxiety is limited by publication bias, and effectiveness for procedural anxiety is moderated by age of participant and mechanism of effect. As a distinctive feature of panoramic meta-analysis, it was demonstrated that effects in procedural anxiety generalize across currently tested indications, while indication explains a relatively low amount of variance in general health anxiety outcomes; but that generalizability in a future trial is highly uncertain. Put otherwise, the evidence for the effectiveness of XR interventions in currently assessed indications cannot be used to assert that XR interventions would likely be effective for *any* given indication.

### Implications for Research and Practice

Although positive effects emerged across a range of general and procedural health anxieties, various indications remain poorly established. Indeed, most meta-analyses are based on context-dependent intervention data, which relates to specific medical procedures (eg, needle insertions and dental surgery) or long-term conditions (eg, cancer and cardiovascular disease). Panoramic meta-analysis methodologies were specifically undertaken to synthesize evidence from diverse clinical contexts and to establish the exchangeability of effects between different intervention types, technologies, populations, and indications. However, key questions remain about the effectiveness of XR within broader domains. For instance, studies are notably lacking in relation to imaging and diagnostic screening procedures (eg, endoscopies and mammography), where anxiety can have well-documented impacts on the quality of care [36-38]. Moreover, most current trials concern pediatric patient groups, despite health-related anxieties showing significant (and potentially growing) prevalence among adults [39,40]. Given the uncertain generalizability of study data that was highlighted in the present analyses, research needs to ascertain the best-suited clinical contexts for future XR interventions.

For the management of procedural anxieties, meta-regressions showed XR to be more effective in children (vs adults), and when using nonimmersive (as opposed to immersive) technologies. These findings imply that age and technology-related variables could influence the success of future XR programs. However, these findings did not replicate general health anxieties, indicating a role for broader, psychopathological factors in shaping effectiveness. For instance, positive outcomes may be more easily achieved when targeting relatively minor forms of anxiety (eg, a child’s worries about needles), compared with more complex and longstanding dispositions (eg, anxieties relating to major surgery or lifelong phobias). Relatedly, nonimmersive XR may be used for implementing simple, distraction-based interventions, whereas immersive devices may be required when attempting to administer more sophisticated or wide-ranging therapeutic solutions that are challenging to deliver successfully. When developing future applications, practitioners must consider the role of these interrelated components, since the value of key technological features (eg, fidelity and face validity) will depend on an intervention’s wider situational context.

Future research is needed to advance our mechanistic understanding of the empirical data. Here, studies should not just explore population- and technology-specific variables (eg, the age of patients, system immersion, and types of hardware), but also broader methodological components relating to how the intervention is delivered and how it actually “works”. Notably, the present results found no significant differences in the management of procedural anxiety between interventions using relaxation or distraction and interventions using education or exposure. However, there were a limited number of trials that examined exposure- and education-based XR methods in the evaluated review evidence, and there were insufficient data for analyzing general health anxiety outcomes. In addition, the included studies rarely provided sufficient details or rationale about their XR procedures and any cointerventions that were present, which limited capabilities for comparing different mechanisms of action. It is important that the contrasting psychophysiological processes implicated by different XR methods are considered during prospective assessments, as they are likely to impact on system requirements. For instance, a high degree of affective fidelity may be important for exposure therapies aiming to provoke “lifelike” stressors and psychophysiological responses; whereas aspects of user presence and task engagement may be more pertinent in simple distraction-based applications (see [41] for related framework). Nonetheless, without further empirical attention in this area, the relationships between different intervention types, psychophysiological mechanisms, and system features remain somewhat unclear. Overall, it is therefore vital that future work aims to address these “gaps” in theoretical understanding, so that the prospective design of XR programs can be sufficiently optimized for clinical implementation.

### Strengths and Limitations

The use of panoramic meta-analysis meant that it was not possible to examine questions not generally addressed in single-indication meta-analyses. While the use of an overview of reviews was efficient in this analysis (and in keeping with the original implementation of this method), it is possible that very recently published trials not captured in existing systematic reviews were missed. In addition, this analysis was reliant on the quality of underpinning systematic reviews including search, extraction, and appraisal. None of the available reviews were rated as high-quality on AMSTAR-2, and many reviews were rated as critically low. Most trials were not rated as being at low RoB in their “parent” reviews. Relatedly, both the trials and reviews contained inconsistencies and inaccuracies in the reporting of study interventions (eg, in terms of describing levels of system immersion, or conceptualizing procedural and general health anxieties). Though included reviews were able to be traced to “saturation”, ensuring the identification of all relevant trials identified across reviews, it is possible that relevant trials were still missed.

### Conclusions

While XR interventions are promising for both procedural and health anxiety, a number of unanswered questions remain. Future research should broaden the indications in which XR interventions are used, and should continue to develop emerging approaches, such as immersive technology interventions, to optimize their effectiveness while continuing to refine approaches for which effectiveness is well established.

## Appendix

Multimedia Appendix 1 Search methods.

Multimedia Appendix 2 List of included reviews.

Multimedia Appendix 3 Extracted study data.

Multimedia Appendix 4 Quality appraisals.

Multimedia Appendix 5 Funnel plots.

Multimedia Appendix 6 PRISMA (Preferred Reporting Items for Systematic Reviews and Meta-Analyses) checklist.

## Abbreviations

AMSTAR-2: A Measurement Tool to Assess Systematic Reviews (Revised Instrument)
PRISMA: Preferred Reporting Items for Systematic Reviews and Meta-Analyses
RoB: risk of bias
XR: extended reality

## Data Availability

The datasets analyzed during this study are available on the Open Science Framework repository [10].
