# Supplementary material for: Extended Reality Interventions for Health and Procedural Anxiety: Panoramic Meta-Analysis Based on Overviews of Reviews
Source: J Med Internet Res. 2025 Jan 8;27:e58086. doi: 10.2196/58086 (PMC11754977; doi:10.2196/58086)
Supplement: Multimedia Appendix 1 [file jmir_v27i1e58086_app1.docx]

**Multimedia Appendix 1.** Search methods

All searches were carried out on 30 May 2023.

**MEDLINE ALL (1946 to May 25, 2023)**

1 exp virtual reality/ 5574

2 Virtual Reality Exposure Therapy/ 877

3 Augmented Reality/ 1137

4 (VR or 'virtual realit*').tw. 21365

5 ('extend* realit*' or XR).tw. 3209

6 (haptic adj2 technolog*).tw. 140

7 (VRCBT or VR-CBT or "virtual exposure" or "immersive technolog*").tw. 197

8 ("automated therap*" or "VR therap*" or "VR cognitive therap*" or "virtual reality therap*" or "virtual reality exposure" or VRET or "virtual reality based exposure" or VRBET).tw. 625

9 ("extended realit*" or "augmented realit*" or "mixed realit*").tw. 4570

10 or/1-9 29424

11 ((phobi* or anxi* or fear*) adj3 (health* or 'procedure*' or 'treatment*' or 'medical')).tw. 22709

12 exp Anxiety/ 110728

13 exp Anxiety Disorders/ 90047

14 exp Phobic Disorders/ 14134

15 11 or 12 or 13 or 14 197719

16 10 and 15 1080

17 review*.ab,ti. or review.pt. or "systematic review"/ or overview*.ab,ti. or meta-analy*.ab,ti. or metaanaly*.ab,ti. or metanaly*.ab,ti. or meta-analysis.pt. or Meta-Analysis/ or meta-regression*.ab,ti. or metaregression*.ab,ti. or (meta adj regression*).ab,ti. or search*.ab. or synthes*.ab,ti. or metasynthes*.ab,ti. or meta-synthes*.ab,ti. or metaethnograph*.ab,ti. or meta-ethnograph*.ab,ti. 5795470

18 (letter or comment or editorial).pt. 2161472

19 17 not 18 5719251

20 16 and 19 261

21 limit 20 to yr="2013 -Current" 183

**Embase (1974 to 2023 May 25)**

1 exp virtual reality/ 25957

2 Virtual Reality Exposure Therapy/ 929

3 Augmented Reality/ 2247

4 (VR or 'virtual realit*').tw. 29372

5 ('extend* realit*' or XR).tw. 6937

6 (haptic adj2 technolog*).tw. 167

7 (VRCBT or VR-CBT or "virtual exposure" or "immersive technolog*").tw. 229

8 ("automated therap*" or "VR therap*" or "VR cognitive therap*" or "virtual reality therap*" or "virtual reality exposure" or VRET or "virtual reality based exposure" or VRBET).tw. 830

9 ("extended realit*" or "augmented realit*" or "mixed realit*").tw. 5464

10 or/1-9 51901

11 ((phobi* or anxi* or fear*) adj3 (health* or 'procedure*' or 'treatment*' or 'medical')).tw. 31606

12 exp Anxiety/ 287499

13 exp Anxiety Disorder/ 315140

14 exp Phobia/ 36664

15 11 or 12 or 13 or 14 559908

16 10 and 15 3402

17 review*.ab,ti. or review.pt. or "systematic review"/ or overview*.ab,ti. or meta-analy*.ab,ti. or metaanaly*.ab,ti. or metanaly*.ab,ti. or meta-analysis.pt. or Meta-Analysis/ or meta-regression*.ab,ti. or metaregression*.ab,ti. or (meta adj regression*).ab,ti. or search*.ab. or synthes*.ab,ti. or metasynthes*.ab,ti. or meta-synthes*.ab,ti. or metaethnograph*.ab,ti. or meta-ethnograph*.ab,ti. 6981657

18 (letter or editorial).pt. or ((animal/ or nonhuman/) not exp human/) 8480445

19 17 not 18 6183558

20 16 and 19 911

21 limit 20 to yr="2013 -Current" 605

**APA PsycInfo (1806 to May Week 4 2023)**

1 exp virtual reality/ 11576

2 Virtual Reality Exposure Therapy/ 257

3 Augmented Reality/ 947

4 (VR or 'virtual realit*').tw. 10464

5 ('extend* realit*' or XR).tw. 792

6 (haptic adj2 technolog*).tw. 49

7 (VRCBT or VR-CBT or "virtual exposure" or "immersive technolog*").tw. 197

8 ("automated therap*" or "VR therap*" or "VR cognitive therap*" or "virtual reality therap*" or "virtual reality exposure" or VRET or "virtual reality based exposure" or VRBET).tw. 666

9 ("extended realit*" or "augmented realit*" or "mixed realit*").tw. 1663

10 or/1-9 16662

11 ((phobi* or anxi* or fear*) adj3 (health* or 'procedure*' or 'treatment*' or 'medical')).tw. 19696

12 exp Anxiety/ 89017

13 exp Anxiety Disorders/ 42535

14 exp Phobias/ or Health Anxiety/ 14939

15 11 or 12 or 13 or 14 130603

16 10 and 15 990

17 exp "Systematic Review"/ or (review* or overview* or meta-analy* or metaanaly* or metanaly*).ab,ti. or "Meta Analysis"/ or meta-regression*.ab,ti. or metaregression*.ab,ti. or (meta adj regression*).ab,ti. or search*.ab. or synthes*.ab,ti. or metasynthes*.ab,ti. or meta-synthes*.ab,ti. or metaethnograph*.ab,ti. or meta-ethnograph*.ab,ti. or ("0800" or "0830" or "1200" or "1300").md. 791312

18 (comment reply or editorial or letter or "review book" or "review media" or "review software other").dt. or (electronic collection or dissertation abstract or encyclopedia).pt. 882960

19 17 not 18 591316

20 16 and 19 182

21 limit 20 to yr="2013 -Current" 109

**Epistemonikos**

((title:(VR OR "virtual realit*" OR "virtual exposure*" OR "extended realit*" OR XR OR "haptic technolog*" OR VRCBT OR "immersive technolog*" OR "automated therap*" OR VRBET OR VRET OR "augmented realit*" OR "mixed realit*")) OR (abstract:(VR OR "virtual realit*" OR "virtual exposure*" OR "extended realit*" OR XR OR "haptic technolog*" OR VRCBT OR "immersive technolog*" OR "automated therap*" OR VRBET OR VRET OR "augmented realit*" OR "mixed realit*")) AND (title:(anxiet* OR phobi* OR fear*) OR abstract:(anxiet* OR phobi* OR fear*)))

Limit to Systematic Reviews 141
