# Supplementary material for: Extended Reality Interventions for Health and Procedural Anxiety: Panoramic Meta-Analysis Based on Overviews of Reviews
Source: J Med Internet Res. 2025 Jan 8;27:e58086. doi: 10.2196/58086 (PMC11754977; doi:10.2196/58086)
Supplement: Multimedia Appendix 2 [file jmir_v27i1e58086_app2.docx]

**Multimedia Appendix 2.** List of included reviews

| **Authors** | **Year** | **Article Title** |
| --- | --- | --- |
| Kılıç, A., Brown, A., Aras, I., Hui, R., Hare, J., Hughes, L. D., McCracken, L. | 2021 | Using Virtual Technology for Fear of Medical Procedures: A Systematic Review of the Effectiveness of Virtual Reality-Based Interventions |
| Koo, C. H., Park, J. W., Ryu, J. H., Han, S. H. | 2020 | The effect of virtual reality on preoperative anxiety: A meta-analysis of randomized controlled trials |
| Tas, F. Q., van Eijk, C. A., Staals, L. M., Legerstee, J. S., Dierckx, B. | 2022 | Virtual reality in pediatrics, effects on pain and anxiety: A systematic review and meta-analysis update |
| Simonetti, V., Tomietto, M., Comparcini, D., Vankova, N., Marcelli, S., Cicolini, G. | 2022 | Effectiveness of virtual reality in the management of paediatric anxiety during the peri-operative period: A systematic review and meta-analysis |
| Yan, X., Yan, Y., Cao, M., Xie, W., O'Connor, S., Lee, J. J., Ho, M. H. | 2023 | Effectiveness of virtual reality distraction interventions to reduce dental anxiety in paediatric patients: A systematic review and meta-analysis |
| Lopez-Valverde, N., Fernandez, J., Lopez-Valverde, A., Juan, L., Ramirez, J., Fraile, J., Payo, J., Antona, L., de Sousa, B., Bravo, M. | 2020 | Use of virtual reality for the management of anxiety and pain in dental treatments: Systematic review and meta-analysis |
| Gao, Y., Xu, Y., Liu, N., Fan, L. | 2023 | Effectiveness of virtual reality intervention on reducing the pain, anxiety and fear of needle-related procedures in paediatric patients: A systematic review and meta-analysis |
| Wang, Y., Guo, L., Xiong, X. | 2022 | Effects of Virtual Reality-Based Distraction of Pain, Fear, and Anxiety During Needle-Related Procedures in Children and Adolescents |
| Turan Kavradim, S., Yangoz, S., Ozer, Z. | 2023 | Effectiveness of virtual reality interventions on physiological and psychological outcomes of adults with cardiovascular disease: A systematic review and meta-analysis |
| Lan, X., Tan, Z., Zhou, T., Huang, Z., Wang, C., Chen, Z., Ma, Y., Kang, T., Gu, Y., Wang, D., Huang, Y. | 2023 | Use of Virtual Reality in Burn Rehabilitation: A Systematic Review and Meta-analysis |
| Gava, V., Fialho, H., Calixtre, L., Barbosa, G., Kamonseki, D. | 2022 | Effects of Gaming on Pain-Related Fear, Pain Catastrophizing, Anxiety, and Depression in Patients with Chronic Musculoskeletal Pain: A Systematic Review and Meta-Analysis |
| Xu, N., Chen, S., Liu, Y., Jing, Y., Gu, P. | 2022 | The Effects of Virtual Reality in Maternal Delivery: Systematic Review and Meta-analysis |
| Baradwan, S., Khadawardi, K., Badghish, E., Alkhamis, W., Dahi, A., Abdallah, K., Kamel, M., Sayd, Z., Mohamed, M., Ali, H., Elhalim, A., Mahmoud, M., Mohamed, A., Mohamed, D. , Shama, A., Hagras, A., Ali, H., Abdelhakim, A., Saleh, M., Badawy, M., Bakry, M. | 2022 | The impact of virtual reality on pain management during normal labor: A systematic review and meta-analysis of randomized controlled trials |
| Wu, Y., Wang, N., Zhang, H., Sun, X., Wang, Y., Zhang, Y. | 2023 | Effectiveness of Virtual Reality in Symptom Management of Cancer Patients: A Systematic Review and Meta-Analysis |
| Obrero-Gaitan, E., Cortes-Perez, I., Calet-Fernandez, T., Garcia-Lopez, H., Lopez Ruiz, M., Osuna-Perez, M. | 2022 | Digital and Interactive Health Interventions Minimize the Physical and Psychological Impact of Breast Cancer, Increasing Women's Quality of Life: A Systematic Review and Meta-Analysis |
| Czech, O., Rutkowski, S., Kowaluk, A., Kiper, P., Malicka, I. | 2023 | Virtual reality in chemotherapy support for the treatment of physical functions, fear, and quality of life in pediatric cancer patients: A systematic review and meta-analysis |
| Bu, X., Ng, P., Xu, W., Cheng, Q., Chen, P., Cheng, A., Liu, X. | 2022 | The Effectiveness of Virtual Reality-Based Interventions in Rehabilitation Management of Breast Cancer Survivors: Systematic Review and Meta-analysis |
| Zeng, Y., Zhang, J., Cheng, A., Cheng, H., Wefel, J. | 2019 | Meta-Analysis of the Efficacy of Virtual Reality-Based Interventions in Cancer-Related Symptom Management |
