# Supplementary material for: Extended Reality Interventions for Health and Procedural Anxiety: Panoramic Meta-Analysis Based on Overviews of Reviews
Source: J Med Internet Res. 2025 Jan 8;27:e58086. doi: 10.2196/58086 (PMC11754977; doi:10.2196/58086)
Supplement: Multimedia Appendix 3 [file jmir_v27i1e58086_app3.docx]

**Multimedia Appendix 3.**  Summary of Extracted Study Data

Studies of procedural anxiety.

| **Parent Review** | **Study** | **Indication** | **Sample Population** | **Anxiety Measure** | **XR Mechanism** | **Intervention Details** |
| --- | --- | --- | --- | --- | --- | --- |
| Kılıç *et al.* (2021) | Gujjar *et al.* (2019) | Dental Anxiety | Adults | Modified Dental Anxiety Scale | Exposure and/or Education | Single exposure therapy session, lasting average of 40 min, provided on immersive VR system |
| Kılıç *et al.* (2021) | Van Twillert *et al.* (2007) | Wound/Burns-related Anxiety | Adults | State-Trait Anxiety Inventory | Relaxation and/or Distraction | Single immersive VR experience (SnowWorld game), provided on HMD for the length of wound care procedure (mean duration: 19 min). |
| Kılıç *et al.* (2021) | McSherry *et al.* (2018) | Wound/Burns-related Anxiety | Adults | Verbal Numeric Scale (0-10) | Relaxation and/or Distraction | Single immersive VR experience (SnowWorld game), provided on HMD for the length of wound care procedure (mean duration: 30 min). |
| Kılıç *et al.* (2021) | Lahti *et al.* (2020) | Dental Anxiety | Adults | Modified Dental Anxiety Scale | Relaxation and/or Distraction | Single non-immersive VR video providing natural scenery experience during procedure (lasting 1 to 3.5 min). |
| Kılıç *et al.* (2021) | Gold & Mahrer (2018) | Needle-related Anxiety | Children | Childhood’s Anxiety Sensitivity Test | Relaxation and/or Distraction | Single immersive experience (Bear Blast game), provided on VR goggles during procedure (lasting approximately 5 min). |
| Kılıç *et al.* (2021) | Jiang *et al.* (2020) | Needle-related Anxiety | Adults | Modified Dental Anxiety Scale | Exposure and/or Education | Single exposure therapy simulation, lasting an average of 90 min, provided on HMD-based immersive VR system. |
| Kılıç *et al.* (2021) | Nunna *et al.* (2019) | Dental Anxiety | Children | Venham’s Clinical Anxiety Rating Scale | Relaxation and/or Distraction | Single non-immersive cartoon video experience, provided in VR glasses during provision of local anaesthetic. |
| Kılıç *et al.* (2021) | Niharika *et al.* (2018) | Dental Anxiety | Children | Modified Child Dental Anxiety Scale | Relaxation and/or Distraction | Two non-immersive cartoon video experiences, lasting approximately 45 min each, provided in HMD-based system. |
| Kılıç *et al.* (2021) | Gerçeker- *et al.* (2020: group 1) | Needle-related Anxiety | Children | The Children’s Anxiety Meter | Relaxation and/or Distraction | Single non-immersive rollercoaster experience, provided via 360° video technology in HMD during blood draw procedure. |
| Kılıç *et al.* (2021) | Gerçeker *et al.* (2020: group 2) | Needle-related Anxiety | Children | The Children’s Anxiety Meter | Relaxation and/or Distraction | Single non-immersive natural scenery experience (Ocean Rift application), provided on HMD during blood draw procedure. |
| Kılıç *et al.* (2021) | Aminabadi *et al.* (2012) | Dental Anxiety | Children | Modified Child Dental Anxiety Scale | Relaxation and/or Distraction | Single non-immersive cartoon experience, provided on VR glasses during dental care procedure (lasting ~30 min). |
| Koo *et al.* (2020) | Bekelis *et al.* (2017) | Surgical Operations (non-dental) | Adults | Amsterdam Preoperative Anxiety and Information score | Exposure and/or Education | Provision of a 360° educational video, lasting 5 min, on HMD-based VR system. Video provided non-immersive information about pre- and post-operative patient experience. |
| Koo *et al.* (2020) | Haisely *et al.* (2020) | Surgical Operations (non-dental) | Adults | Numeric Rating Scale | Relaxation and/or Distraction | Single mindfulness-based relaxation experience (lasting ~28 min) provided on immersive HMD-based VR system. |
| Koo *et al.* (2020) | Noben *et al.* (2019) | Surgical Operations (non-dental) | Adults | Visual Analogue Scale | Exposure and/or Education | Provision of a non-immersive 360° information video. Presented to patients on an HMD-based VR system. |
| Koo *et al.* (2020) | Robertson *et al.* (2017) | Surgical Operations (non-dental) | Adults | Hospital Anxiety and Depression Scale | Relaxation and/or Distraction | Single non-immersive natural scenery experience (Perfect Beach application), provided via 360° video on HMD for 9-min. |
| Koo *et al.* (2020) | Yang *et al.* (2019) | Surgical Operations (non-dental) | Adults | Amsterdam Preoperative Anxiety and Information score | Exposure and/or Education | Presentation of 3-D MRI scan images via immersive HMD-based VR system. |
| Koo *et al.* (2020) | Dehghan *et al.* (2019) | Surgical Operations (non-dental) | Children | Yale Preoperative Anxiety Scale | Exposure and/or Education | Single non-immersive operating room experience, provided via VR glasses and PC screen (exposure time: 5 min). |
| Koo *et al.* (2020) | Eijlers *et al.* (2019) | Surgical Operations (non-dental) | Children | Modified Yale Preoperative Anxiety Scale | Exposure and/or Education | Single immersive operating room experience, provided via HMD-based VR system (exposure time: ~15 min). |
| Koo *et al.* (2020) | Ryu *et al.* (2017) | Surgical Operations (non-dental) | Children | Modified Yale Preoperative Anxiety Scale | Exposure and/or Education | Single non-immersive 360° video experience, providing a 4-min tour of the operating room (via HMD-based VR system). |
| Koo *et al.* (2020) | Ryu *et al.* (2018) | Surgical Operations (non-dental) | Children | Modified Yale Preoperative Anxiety Scale | Exposure and/or Education | Provision of a 5-min immersive VR educational experience. Experience gave information about the operative process. |
| Koo *et al.* (2020) | Ryu *et al.* (2019) | Surgical Operations (non-dental) | Children | Modified Yale Preoperative Anxiety Scale | Exposure and/or Education | Single non-immersive 360° video experience, providing a 4-min tour of the operating room (via HMD-based VR system). |
| Tas *et al.* (2022) | Chan *et al.* (2019: group 1) | Needle-related Anxiety | Children | Visual Analogue Thermometer | Relaxation and/or Distraction | Single immersive experience (Underwater adventure game), provided on VR system during needle-based procedure (lasting an average of 7 min). |
| Tas *et al.* (2022) | Chan *et al.* (2019: group 2) | Needle-related Anxiety | Children | Visual Analogue Thermometer | Relaxation and/or Distraction | Single immersive experience (Underwater adventure game), provided on VR system during needle-based procedure (lasting an average of 5 min). |
| Tas *et al.* (2022) | Dumoulin *et al.* (2019) | Needle-related Anxiety | Children | Visual Analog Scale | Relaxation and/or Distraction | Single immersive experience (custom-made interactive game), provided on HMD-based VR system during needle-based procedure (lasting approximately 10 min). |
| Tas *et al.* (2022) | Jung *et al.* (2020) | Surgical Operations (non-dental) | Children | Modified Yale Preoperative Anxiety Scale | Relaxation and/or Distraction | Single immersive experience (interactive game), performed on VR system during the provision of anaesthetic. |
| Tas *et al.* (2022) | Liu *et al.* (2020) | Surgical Operations (non-dental) | Children | Subjective Units of Distress anxiety score | Relaxation and/or Distraction | Single immersive experience (SpaceBurgers game), provided on HMD-based VR system during medical operation. |
| Tas *et al.* (2022) | Schneider *et al.* (1999) | Surgical Operations (non-dental) | Children | State-Trait Anxiety Inventory for Children | Relaxation and/or Distraction | Single immersive experience (one of three commercially available games), provided on VR glasses during surgical procedure. |
| Tas *et al.* (2022) | Gershon *et al.* (2004) | Surgical Operations (non-dental) | Children | Visual Analogue Scale | Relaxation and/or Distraction | Single immersive zoo experience, provided on HMD-based VR system during procedure (lasting 5-10 min). |
| Tas *et al.* (2022) | Wolitzky *et al.* (2005) | Surgical Operations (non-dental) | Children | Visual Analogue Scale for Pain and Anxiety | Relaxation and/or Distraction | Single immersive zoo experience, provided on HMD-based VR system during port access procedure. |
| Tas *et al.* (2022) | Piskorz & Czub (2017) | Needle-related Anxiety | Children | Visual Analogue Scale | Relaxation and/or Distraction | Single immersive VR experience (multiple object tracking game), provided on HMD-based system during procedure (lasting approximately 5 min). Prior to this, patients received 15 min of training on VR device. |
| Tas *et al.* (2022) | Han *et al.* (2019) | Imaging-related Anxiety | Children | Observational Scale of Behavioural Distress for radiographic procedures | Exposure and/or Education | Single non-immersive operating room experience, lasting 3 min, provided via 360° video on HMD-based system. |
| Simonetti *et al.* (2022) | Park *et al.* (2019) | Surgical Operations (non-dental) | Children | Modified Yale Preoperative Anxiety Scale | Exposure and/or Education | Single non-immersive 360° video experience, providing a 4-min tour of the operating room (via HMD-based VR system). |
| Yan *et al.* (2023) | Aditya *et al.* (2021) | Dental Anxiety | Children | Venham’s Picture Test | Relaxation and/or Distraction | Single non-immersive cartoon video experience, presented on HMD-based VR system during provision of local anaesthetic. |
| Yan *et al.* (2023) | Aminabadi *et al.* (2022) | Dental Anxiety | Children | Faces version of the Modified Child Dental Anxiety Scale | Relaxation and/or Distraction | Two non-immersive cartoon video experiences, provided in VR glasses during dental treatment. |
| Yan *et al.* (2023) | Buldur & Candan (2021) | Dental Anxiety | Children | Children’s Fear Survey Schedule-Dental Subscale | Relaxation and/or Distraction | Single non-immersive cartoon video experience, provided on HMD-based VR system during dental procedure (lasting an average of 31 min). |
| Yan *et al.* (2023) | Du *et al.* (2022) | Dental Anxiety | Children | Children’s Fear Survey Schedule-Dental Subscale | Natural Scenery (sea, forest, magic world) | Three immersive natural scenery experiences, lasting under 20 min, provided on HMD-based VR system. |
| Yan *et al.* (2023) | Gomez-Polo *et al.* (2021) | Dental Anxiety | Children | Facial Image Scale | Relaxation and/or Distraction | Non-immersive cartoon video experience, provided on VR glasses. |
| Yan *et al.* (2023) | Greeshma *et al.* (2021) | Dental Anxiety | Children | Facial Image Scale | Relaxation and/or Distraction | Non-immersive roller coaster experience, provided on HMD-based VR system. |
| Yan *et al.* (2023) | Pande *et al.* (2020) | Dental Anxiety | Children | Facial Image Scale | Relaxation and/or Distraction | Single non-immersive cartoon video experience, provided on VR Box system during the course of dental treatment. |
| Yan *et al.* (2023) | Ran *et al.* (2021) | Dental Anxiety | Children | Children’s Fear Survey Schedule-Dental Subscale | Relaxation and/or Distraction | Single non-immersive experience (Underwater adventure game), provided on HMD-based VR system during the course of dental treatment (lasting an average of 19 min). |
| Yan *et al.* (2023) | Shetty *et al.* (2019) | Dental Anxiety | Children | Modified Dental Anxiety Scale | Relaxation and/or Distraction | Non-immersive cartoon video, provided on VR glasses before (for 5 min) and during operation (which lasted no more than 45 min). |
| Lopez-Valverde *et al.* (2023) | Mitrakul *et al.* (2015) | Dental Anxiety | Children | FLACC Consolability Scale | Relaxation and/or Distraction | Single non-immersive cartoon video experience, provided on VR glasses during dental treatment (lasting 30-45 min). |
| Lopez-Valverde *et al.* (2023) | Asvanund *et al.* (2015) | Dental Anxiety | Children | FLACC Consolability Scale | Relaxation and/or Distraction | Non-immersive distraction experience, provided on VR glasses. |
| Lopez-Valverde *et al.* (2023) | Al-Khotani *et al.* (2016) | Dental Anxiety | Children | Modified Dental Anxiety Scale | Relaxation and/or Distraction | Single non-immersive cartoon video experience, provided on VR glasses during dental treatment. |
| Gao *et al.* (2023) | Canares *et al.* (2021) | Needle-related Anxiety | Children and Young Adults | Children's Anxiety Meter-State | Relaxation and/or Distraction | Single immersive VR experience (from selection of miscellaneous games), provided on HMD-based system for average of 11 min. |
| Gao *et al.* (2023) | Gold *et al.* (2021) | Needle-related Anxiety | Children | Visual Analog Scale (0-10 thermometer) | Relaxation and/or Distraction | Single immersive experience (Bear Blast game), provided on HMD-based VR system before (< 5 min) and during subsequent PIVC procedure. |
| Gao *et al.* (2023) | Goldman & Behboudi (2021a) | Needle-related Anxiety | Children | Venham Situational Anxiety Scale | Relaxation and/or Distraction | Single non-immersive rollercoaster experience, provided via 360° video technology in HMD during intravenous catheterization procedure (lasting average of 5 min) and for up to 15 mins afterwards. |
| Gao *et al.* (2023) | Goldman & Behboudi (2021b) | Needle-related Anxiety | Children | Venham Situational Anxiety Scale | Relaxation and/or Distraction | Single non-immersive rollercoaster experience, provided via 360° video technology in HMD during laceration repair procedure (lasting average of 27 min) and for up to 15 mins afterwards. |
| Gao *et al.* (2023) | Hundert *et al.* (2021) | Needle-related Anxiety | Children | Numerical Rating Scale | Relaxation and/or Distraction | Single immersive experience (Underwater adventure game), provided on HMD-based VR system before, during, and after subcutaneous port needle insertion |
| Gao *et al.* (2023) | İnangil *et al.* (2020) | Needle-related Anxiety | Children | Child Fear Scale | Relaxation and/or Distraction | Single non-immersive 360° cartoon video experience, provided on HMD-based VR system during needle procedure (lasting around 4 mins). |
| Gao *et al.* (2023) | Litwin *et al.* (2021) | Needle-related Anxiety | Children | Numerical Rating Scale | Relaxation and/or Distraction | Single immersive experience (Underwater adventure game), provided on HMD-based VR system. |
| Gao *et al.* (2023) | Gerçeker- *et al.* (2021) | Needle-related Anxiety | Children | Children's Anxiety Meter-State | Relaxation and/or Distraction | Single non-immersive experience (one of three commercially available simulations), provided on HMD-based VR system for 5-8 min. |
| Wang *et al.* (2022) | Koc Ozkan & Polat (2020) | Needle-related Anxiety | Children | Visual Analog Scale | Relaxation and/or Distraction | Single non-immersive distraction experience, provided via 360° video technology in VR glasses during venipuncture procedure. |

Studies of General Health Anxiety

| **Parent Review** | **Study** | **Indication** | **Sample Population** | **Anxiety Measure** | **XR Mechanism** | **Intervention Details** |
| --- | --- | --- | --- | --- | --- | --- |
| Turan-Kavradim *et al.* (2023) | Hessabi *et al.* (2020) | Cardiovascular Disease | Adults | State-Trait Anxiety Inventory | Relaxation and/or Distraction | Two non-immersive natural scenery experiences, lasting 15 min, provided via 360° videos on an HMD-based VR system. |
| Turan-Kavradim *et al.* (2023) | Jóźwik *et al.* (2021a) | Cardiovascular Disease | Adults | Hospital Anxiety and Depression Scale | Relaxation and/or Distraction | Eight sessions involving immersive relaxation experiences (TierOne Multisensory application), provided three times a week on an HMD-based VR system. |
| Turan-Kavradim *et al.* (2023) | Jóźwik *et al.* (2021b) | Cardiovascular Disease | Adults | Hospital Anxiety and Depression Scale | Relaxation and/or Distraction | Eight sessions involving immersive relaxation experiences (TierOne Multisensory application), provided three times a week on an HMD-based VR system. |
| Turan-Kavradim *et al.* (2023) | Keshvari *et al.* (2021) | Cardiovascular Disease | Adults | State-Trait Anxiety Inventory | Relaxation and/or Distraction | Single non-immersive natural scenery experience, lasting 5 min, provided via 360° video on an HMD-based system. |
| Turan-Kavradim *et al.* (2023) | Maciolek *et al.* (2020) | Cardiovascular Disease | Adults | State‑Trait Anxiety Inventory | Relaxation and/or Distraction | Eight 20-min sessions, involving non-immersive natural scenery VR experiences (with music). |
| Turan-Kavradim *et al.* (2023) | Morgan *et al.* (2021) | Cardiovascular Disease | Adults | State-Trait Anxiety Inventory | Exposure and/or Education | Provision of a 360° educational video, lasting 10 min, on HMD-based VR system. Video provided non-immersive information about the operative patient experience. |
| Turan-Kavradim *et al.* (2023) | Szczepańska-Gieracha *et al.* (2021) | Cardiovascular Disease | Adults | Hospital Anxiety and Depression Scale | Relaxation and/or Distraction | Eight 20-min sessions involving immersive relaxation experiences (TierOne Multisensory application). Provided on an HMD-based VR system, twice a week for four weeks. |
| Lan *et al.* (2023) | Hoffman *et al.* (2000) | Wound/Burns-related | Adults | Visual Analogue Scale | Relaxation and/or Distraction | Immersive experience (Spider World game), provided on an HMD-based VR system during physical therapy session. |
| Lan *et al.* (2023) | Hoffman *et al.* (2001) | Wound/Burns-related | Adults and Children | Visual Analogue Scale (rating of 0-10) | Relaxation and/or Distraction | Three sessions involving immersive VR experiences (Spider World and Snow World games), provided on an HMD-based VR system for around 3-6 min. |
| Lan *et al.* (2023) | Morris *et al.* (2010) | Wound/Burns-related | Adults | Burn Specific Pain Anxiety Scale | Relaxation and/or Distraction | Immersive experience (Chicken Little game), provided on an HMD- and joystick-based VR system (lasting up to 10 mins). |
| Gava *et al.* (2022) | Tejera *et al.* (2020) | Chronic Pain | Adults | Pain Anxiety Symptoms Scale | Relaxation and/or Distraction | Eight non-immersive physical therapy sessions (using Vox Play exergame). Provided on a smartphone-based HMD system, twice a week for four weeks. |
| Xu *et al.* (2022) | Akin *et al.* (2021) | Maternity | Adults | Perinatal Anxiety Screening Scale | Relaxation and/or Distraction | Provision of scanning images during delivery. Presented for an average of 14 min on non-immersive VR Glasses system. |
| Xu *et al.* (2022) | Li *et al.* (2020) | Maternity | Adults | Self-rating Anxiety Scale | Relaxation and/or Distraction | Non-immersive natural scenery experience, provided on VR glasses device from first stage labour until completion. |
| Xu *et al.* (2022) | Lin *et al.* (2021) | Maternity | Adults | Self-rating Anxiety Scale | Relaxation and/or Distraction | Non-immersive natural scenery experience, provided on VR glasses device for 5-60 mins. |
| Xu *et al.* (2022) | Liu & Wan (2020) | Maternity | Adults | Self-rating Anxiety Scale | Unclear | Self-selected VR activity involving non-immersive experience. Provided on HMD-based system for up to 2h per day during pregnancy and delivery. |
| Xu *et al.* (2022) | Momenyan *et al.* (2021) | Maternity | Adults | Numerical rating scale (non-specific) | Relaxation and/or Distraction | Two 10-min sessions involving non-immersive natural scenery experiences. Provided during labour via 360° videos on an HMD-based VR system. |
| Baradwan *et al.* (2022) | Frey *et al.* (2019) | Maternity | Adults | Numerical rating scale (non-specific) | Relaxation and/or Distraction | Single immersive natural scenery experience (Ocean Rift application), provided during labour for 10 mins on HMD-based system. |
| Wu *et al.* (2023) | Gao *et al.* (2022) | Cancer | Adults | State-Trait Anxiety Inventory | Exposure and/or Education | Immersive educational tool, lasting 30 mins. Provided information about radiotherapy on an HMD-based VR system. |
| Wu *et al.* (2023) | Mohammad & Ahmad (2019) | Cancer | Adults | State Anxiety Inventory | Relaxation and/or Distraction | Single immersive natural scenery experience (Ocean Rift application), provided alongside morphine during breast cancer treatment. |
| Wu *et al.* (2023) | Tennant *et al.* (2020) | Cancer | Children | Visual Analogue Scale | Relaxation and/or Distraction | Single non-immersive natural scenery experience, lasting 10 mins, provided via 360° videos on an HMD-based VR system. |
| Wu *et al.* (2023) | Turrado *et al.* (2021) | Cancer | Adults | State-Trait Anxiety Inventory | Exposure and/or Education | Single 17-min VR session, which exposed users to the pre-, peri-, and post-operation patient experience. Provided via 360° videos on a non-immersive HMD-based system. |
| Wu *et al.* (2023) | Wong *et al.* (2020) | Cancer | Children | Chinese Version of the State Anxiety Scale for Children | Relaxation and/or Distraction | Single non-immersive video experience (of either cartoons or natural scenery content). Provided on VR system from before cannulation until end of the procedure. |
| Wu *et al.* (2023) | Zhang *et al.* (2022) | Cancer | Adults | Self-Rating Anxiety Scale | Relaxation and/or Distraction | Six 30-min sessions involving immersive natural scenery experiences. Provided on an HMD-based VR system, over 3-month period of care. |
| Obrero-Gaitan *et al.* (2022) | Chirico *et al.* (2020) | Cancer | Adults | State Anxiety Inventory | Relaxation and/or Distraction | Single natural scenery experience (Second Life application), provided for 20 min on an immersive VR system. |
| Obrero-Gaitan *et al.* (2022) | Jimenez *et al.* (2018) | Cancer | Adults | State-Trait Anxiety Inventory | Exposure and/or Education | Eighteen 60-min sessions involving immersive educational tool. Provided realistic sensorimotor stimuli and patient-directed information about radiotherapy. |
| Czech *et al.* (2023) | Sharifpour *et al.* (2021) | Cancer | Children | Pain anxiety symptoms scale | Relaxation and/or Distraction | Single non-immersive natural scenery experience, lasting 30 mins. Provided during chemotherapy, via 360° videos on an HMD-based VR system. |
| Bu *et al.* (2022) | Buche *et al.* (2021: group 1) | Cancer | Adults | State Anxiety Inventory | Relaxation and/or Distraction | Repeated 30-min sessions involving natural scenery experiences (Nature Treks application). Used ‘contemplative’ non-immersive mode of simulation over period of 10 months. |
| Bu *et al.* (2022) | Buche *et al.* (2021: group 2) | Cancer | Adults | State Anxiety Inventory | Relaxation and/or Distraction | Repeated 30-min sessions involving natural scenery experiences (Nature Treks application). Used ‘participatory’ immersive mode of simulation over period of 10 months. |
| Zeng *et al.* (2019) | Baños *et al.* (2013) | Cancer | Adults | Visual Analogue Scale item (rated 1-7) | Relaxation and/or Distraction | Four 30-min sessions involving non-immersive natural scenery and relaxation experiences. Provided on monitor-based VR system, during 1 week of care. |
| Zeng *et al.* (2019) | Glennon *et al.* (2018) | Cancer | Adults | Visual Analogue Scale (5 item scale) | Relaxation and/or Distraction | Single non-immersive natural scenery experience, lasting an average of 15 mins. Provided on VR goggles during treatment procedures. |

References

Aditya, P., Prasad, M. G., Nagaradhakrishna, A., Raju, N. S., & Babu, D. N. (2021). Comparison of effectiveness of three distraction techniques to allay dental anxiety during inferior alveolar nerve block in children: A randomized controlled clinical trial. *Heliyon, 7(9)*.

Al-Khotani, A., Bello, L. A. a., & Christidis, N. (2016). Effects of audiovisual distraction on children’s behaviour during dental treatment: a randomized controlled clinical trial. *Acta Odontologica Scandinavica, 74(6),* 494-501.

Aminabadi, N. A., Erfanparast, L., Sohrabi, A., Oskouei, S. G., & Naghili, A. (2012). The impact of virtual reality distraction on pain and anxiety during dental treatment in 4-6 year-old children: a randomized controlled clinical trial. *Journal of Dental Research, Dental Clinics, Dental Prospects, 6(4),* 117.

Aminabadi, N. A., Golsanamlou, O., Halimi, Z., & Jamali, Z. (2022). Assessing the different levels of virtual reality that influence anxiety, behavior, and oral health status in preschool children: Randomized controlled clinical trial. *JMIR Perioperative Medicine, 5(1),* e35415.

Asvanund, Y., Mitrakul, K., Juhong, R-O., & Arunakul, M. (2015). Effect of audiovisual eyeglasses during local anesthesia injections in 5-to 8-year-old children. *Quintessence International, 46(6)*.

Baños, R., Espinoza, M., García-Palacios, A., Cervera, J. M., Esquerdo, G., Barrajón, E., & Botella, C. (2013). A positive psychological intervention using virtual reality for patients with advanced cancer in a hospital setting: a pilot study to assess feasibility. *Supportive Care in Cancer, 21,* 263-270.

Baradwan, S., Khadawardi, K., Badghish, E., Alkhamis, W., Dahi, A., Abdallah, K., Kamel, M., Sayd, Z., Mohamed, M., Ali, H., Elhalim, A., Mahmoud, M., Mohamed, A., Mohamed, D., Shama, A., Hagras, A., Ali, H., Abdelhakim, A., Saleh, M., . . . Bakry, M. (2022). The impact of virtual reality on pain management during normal labor: A systematic review and <i>meta</i>-analysis of randomized controlled trials. *Sexual & Reproductive Healthcare, 32,* 100720.

Bekelis, K., Calnan, D., Simmons, N., MacKenzie, T., & Kakoulides, G. (2017). Effect of an Immersive Preoperative Virtual Reality Experience on Patient Reported Outcomes: *A Randomized Controlled Trial. Annals of Surgery, 265(6),* 1068-1073.

Bu, X., Ng, P. H. F., Xu, W., Cheng, Q., Chen, P. Q., Cheng, A. S. K., & Liu, X. (2022). The Effectiveness of Virtual Reality–Based Interventions in Rehabilitation Management of Breast Cancer Survivors: Systematic Review and Meta-analysis. *JMIR Serious Games, 10(1),* e31395.

Buche, H., Michel, A., Piccoli, C., & Blanc, N. (2021). Contemplating or acting? Which immersive modes should be favored in virtual reality during physiotherapy for breast cancer rehabilitation. *Frontiers in Psychology, 12*, 631186.

Buldur, B., & Candan, M. (2020). Does virtual reality affect children’s dental anxiety, pain, and behaviour? a randomised, placebo-controlled, cross-over trial. *Pesquisa Brasileira em Odontopediatria e Clínica Integrada, 21*, e0082.

Canares, T., Parrish, C., Santos, C., Badawi, A., Stewart, A., Kleinman, K., Psoter, K., & McGuire, J. (2021). Pediatric coping during venipuncture with virtual reality: Pilot randomized controlled trial. *JMIR Pediatrics and Parenting, 4(3),* e26040.

Chan, E., Hovenden, M., Ramage, E., Ling, N., Pham, J. H., Rahim, A., Lam, C., Liu, L., Foster, S., & Sambell, R. (2019). Virtual reality for pediatric needle procedural pain: two randomized clinical trials. *The Journal of Pediatrics, 209,* 160-167.

Chirico, A., Maiorano, P., Indovina, P., Milanese, C., Giordano, G. G., Alivernini, F., Iodice, G., Gallo, L., De Pietro, G., & Lucidi, F. (2020). Virtual reality and music therapy as distraction interventions to alleviate anxiety and improve mood states in breast cancer patients during chemotherapy. *Journal of Cellular Physiology, 235(6),* 5353-5362.

Czech, O., Rutkowski, S., Kowaluk, A., Kiper, P., & Malicka, I. (2023). Virtual reality in chemotherapy support for the treatment of physical functions, fear, and quality of life in pediatric cancer patients: A systematic review and meta-analysis. *Frontiers in Public Health, 11*.

Dehghan, F., Jalali, R., & Bashiri, H. (2019). The effect of virtual reality technology on preoperative anxiety in children: a Solomon four-group randomized clinical trial. *Perioperative Medicine, 8,* 1-7.

Du, Q., Ma, X., Wang, S., Zhou, S., Luo, C., Tian, K., Fei, W., & Liu, X. (2022). A digital intervention using virtual reality helmets to reduce dental anxiety of children under local anesthesia and primary teeth extraction: A randomized clinical trial. *Brain and Behavior, 12(6),* e2600.

Dumoulin, S., Bouchard, S., Ellis, J., Lavoie, K. L., Vézina, M-P., Charbonneau, P., Tardif, J., & Hajjar, A. (2019). A randomized controlled trial on the use of virtual reality for needle-related procedures in children and adolescents in the emergency department. *Games for Health, 8(4),* 285-293.

Eijlers, R., Utens, E. M. W. J., Staals, L. M., de Nijs, P. F. A., Berghmans, J. M., Wijnen, R. M. H., Hillegers, M. H. J., Dierckx, B., & Legerstee, J. S. (2019). Systematic Review and Meta-analysis of Virtual Reality in Pediatrics: Effects on Pain and Anxiety. *Anesthesia and Analgesia, 129(5),* 1344-1353.

Frey, D. P., Bauer, M. E., Bell, C. L., Low, L. K., Hassett, A. L., Cassidy, R. B., Boyer, K. D., & Sharar, S. R. (2019). Virtual reality analgesia in labor: the VRAIL pilot study—a preliminary randomized controlled trial suggesting benefit of immersive virtual reality analgesia in unmedicated laboring women. *Anesthesia & Analgesia, 128(6),* e93-e96.

Gao, J., Liu, S., Zhang, S., Wang, Y., Liang, Z., Feng, Q., Hu, M., & Zhang, Q. (2022). Pilot study of a virtual reality educational intervention for radiotherapy patients prior to initiating treatment. *Journal of Cancer Education,* 1-8.

Gao, Y., Xu, Y., Liu, N., & Fan, L. (2023). Effectiveness of virtual reality intervention on reducing the pain, anxiety and fear of needle‐related procedures in paediatric patients: A systematic review and meta‐analysis. *Journal of Advanced Nursing, 79(1),* 15-30.

Gava, V., Fialho, H., Calixtre, L., Barbosa, G., & Kamonseki, D. (2022). Effects of Gaming on Pain-Related Fear, Pain Catastrophizing, Anxiety, and Depression in Patients with Chronic Musculoskeletal Pain: A Systematic Review and Meta-Analysis. *Games for Health*, *11(6),* 369-384.

Gerçeker, G. Ö., Ayar, D., Özdemir, E. Z., & Bektaş, M. (2020). Effects of virtual reality on pain, fear and anxiety during blood draw in children aged 5–12 years old: A randomised controlled study. *Journal of Clinical Nursing, 29(7-8),* 1151-1161.

Gerçeker, G. Ö., Bektaş, M., Aydınok, Y., Ören, H., Ellidokuz, H., & Olgun, N. (2021). The effect of virtual reality on pain, fear, and anxiety during access of a port with huber needle in pediatric hematology-oncology patients: Randomized controlled trial. *European Journal of Oncology Nursing, 50,* 101886.

Gershon, J., Zimand, E., Pickering, M., Rothbaum, B. O., & Hodges, L. (2004). A pilot and feasibility study of virtual reality as a distraction for children with cancer. *Journal of the American Academy of Child & Adolescent Psychiatry, 43(10),* 1243-1249.

Glennon, C., McElroy, S. F., Connelly, L. M., Lawson, L. M., Bretches, A. M., Gard, A. R., & Newcomer, L. R. (2018). Use of Virtual Reality to Distract From Pain and Anxiety. In *Oncology Nursing Forum* (Vol. 45, No. 4).

Gold, J. I., & Mahrer, N. E. (2018). Is virtual reality ready for prime time in the medical space? A randomized control trial of pediatric virtual reality for acute procedural pain management*. Journal of Pediatric Psychology, 43(3),* 266-275.

Gold, J. I., SooHoo, M., Laikin, A. M., Lane, A. S., & Klein, M. J. (2021). Effect of an immersive virtual reality intervention on pain and anxiety associated with peripheral intravenous catheter placement in the pediatric setting: a randomized clinical trial. *JAMA Network Open, 4(8),* e2122569-e2122569.

Goldman, R. D., & Behboudi, A. (2021a). Virtual reality for intravenous placement in the emergency department—a randomized controlled trial. *European Journal of Pediatrics, 180,* 725-731.

Goldman, R. D., & Behboudi, A. (2021b). Pilot randomized controlled trial of virtual reality vs. standard-of-care during pediatric laceration repair. *Journal of Child & Adolescent Trauma, 14,* 295-298.

Gómez-Polo, C., Vilches, A., Ribas, D., Castaño-Séiquer, A., & Montero, J. (2021). Behaviour and anxiety management of paediatric dental patients through virtual reality: A randomised clinical trial. *Journal of Clinical Medicine, 10(14),* 3019.

Greeshma, G., George, S., Anandaraj, S., Sain, S., Jose, D., Sreenivas, A., Pillai, G., & Mol, N. (2021). Comparative evaluation of the efficacy of virtual reality distraction, audio distraction and tell-show-do techniques in reducing the anxiety level of pediatric dental patients: An in vivo study. *International Journal of Clinical Pediatric Dentistry,* 14(2), S173.

Gujjar, K. R., van Wijk, A., Kumar, R., & de Jongh, A. (2019). Efficacy of virtual reality exposure therapy for the treatment of dental phobia in adults: A randomized controlled trial. *Journal of Anxiety Disorders, 62,* 100-108.

Haisley, K. R., Straw, O. J., Müller, D. T., Antiporda, M. A., Zihni, A. M., Reavis, K. M., Bradley, D. D., & Dunst, C. M. (2020). Feasibility of implementing a virtual reality program as an adjuvant tool for peri-operative pain control; results of a randomized controlled trial in minimally invasive foregut surgery. *Complementary Therapies in Medicine, 49,* 102356.

Han, S., Park, J., Choi, S., Kim, J., Lee, H., Yoo, H., & Ryu, J. (2019). Effect of immersive virtual reality education before chest radiography on anxiety and distress among pediatric patients: a randomized clinical trial. *JAMA pediatrics, 173(11),* 1026-1031.

Hessabi, M., Sajjadi, M., Shareinia, H., & Rouhani, M. (2020). The effect of virtual reality on anxiety and quality of sleep in patients in cardiac care unit. *International Journal of Pharmaceutical and Phytopharmacological Research, 10*, 37-42.

Hoffman, H. G., Doctor, J. N., Patterson, D. R., Carrougher, G. J., & Furness, T. A. (2000). Virtual reality as an adjunctive pain control during burn wound care in adolescent patients. *Pain, 85,* 305-309.

Hoffman, H. G., Patterson, D. R., Carrougher, G. J., & Sharar, S. R. (2001). Effectiveness of virtual reality–based pain control with multiple treatments. *The Clinical journal of Pain, 17(3),* 229-235.

Hundert, A. S., Birnie, K. A., Abla, O., Positano, K., Cassiani, C., Lloyd, S., Tiessen, P. H., Lalloo, C., Jibb, L. A., & Stinson, J. (2022). A pilot randomized controlled trial of virtual reality distraction to reduce procedural pain during subcutaneous port access in children and adolescents with cancer. *The Clinical journal of Pain, 38(3),* 189-196.

İnangil, D., Şendir, M., & Büyükyılmaz, F. (2020). Efficacy of cartoon viewing devices during phlebotomy in children: a randomized controlled trial. *Journal of PeriAnesthesia Nursing, 35(4),* 407-412.

Jiang, M. Y. W., Upton, E., & Newby, J. M. (2020). A randomised wait-list controlled pilot trial of one-session virtual reality exposure therapy for blood-injection-injury phobias. *Journal of Affective Disorders, 276,* 636-645.

Jimenez, Y. A., Cumming, S., Wang, W., Stuart, K., Thwaites, D. I., & Lewis, S. J. (2018). Patient education using virtual reality increases knowledge and positive experience for breast cancer patients undergoing radiation therapy. *Supportive Care in Cancer, 26,* 2879-2888.

Jóźwik, S., Cieślik, B., Gajda, R., & Szczepańska-Gieracha, J. (2021a). Evaluation of the impact of virtual reality-enhanced cardiac rehabilitation on depressive and anxiety symptoms in patients with coronary artery disease: a randomised controlled trial. *Journal of Clinical Medicine, 10(10),* 2148.

Jóźwik, S., Cieślik, B., Gajda, R., & Szczepańska-Gieracha, J. (2021b). The use of virtual therapy in cardiac rehabilitation of female patients with heart disease. *Medicina, 57(8),* 768.

Jung, M. J., Libaw, J. S., Ma, K., Whitlock, E. L., Feiner, J. R., & Sinskey, J. L. (2020). Pediatric Distraction on Induction of Anesthesia With Virtual Reality and Perioperative Anxiolysis: A Randomized Controlled Trial. *Anesthesia & Analgesia, 132(3),* 798-806.

Keshvari, M., Yeganeh, M. R., Paryad, E., Roushan, Z. A., & Pouralizadeh, M. (2021). The effect of virtual reality distraction on reducing patients' anxiety before coronary angiography: a randomized clinical trial study. *The Egyptian Heart Journal, 73,* 1-8.

Kılıç, A., Brown, A., Aras, I., Hui, R., Hare, J., Hughes, L. D., & McCracken, L. M. (2021). Using Virtual Technology for Fear of Medical Procedures: A Systematic Review of the Effectiveness of Virtual Reality-Based Interventions. *Annals of Behavioral Medicine, 55(11),* 1062-1079.

Koo, C., Park, J., Ryu, J., & Han, S. (2020). The Effect of Virtual Reality on Preoperative Anxiety: A Meta-Analysis of Randomized Controlled Trials*. Journal of Clinical Medicine, 9(10),* 3151.

Lahti, S., Suominen, A., Freeman, R., Lähteenoja, T., & Humphris, G. (2020). Virtual reality relaxation to decrease dental anxiety: Immediate effect randomized clinical trial. *JDR Clinical & Translational Research, 5(4),* 312-318.

Lan, X., Tan, Z., Zhou, T., Huang, Z., Huang, Z., Wang, C., Chen, Z., Ma, Y., Kang, T., Gu, Y., Wang, D., & Huang, Y. (2023). Use of Virtual Reality in Burn Rehabilitation: A Systematic Review and Meta-analysis. *Archives of Physical Medicine and Rehabilitation, 104(3),* 502-513.

Li, Z., Liu, L., & Yin, Y. (2020). Effectiveness of VR glasses in primiparous delivery. *China Modern Med, 27(15),* 246-249.

Litwin, S. P., Nguyen, C., Hundert, A., Stuart, S., Liu, D., Maguire, B., Matava, C., & Stinson, J. (2021). Virtual reality to reduce procedural pain during IV insertion in the pediatric emergency department: a pilot randomized controlled trial. *The Clinical Journal of Pain, 37(2),* 94-101.

Liu, K. Y., Ninan, S. J., Laitman, B. M., Goldrich, D. Y., Iloreta, A. M., & Londino III, A. V. (2020). Virtual reality as distraction analgesia and anxiolysis for pediatric otolaryngology procedures. *The Laryngoscope, 131(5),* e1714-e1721.

Liu, X., & Wan, L. (2020). Effectiveness of virtual reality technology in reducing pain and anxiety during natural childbirth in primiparous women. *Chinese General Practice Nursing, 18(21),* 2668-2670.

López-Valverde, N., Muriel Fernández, J., López-Valverde, A., Valero Juan, L. F., Ramírez, J. M., Flores Fraile, J., Herrero Payo, J., Blanco Antona, L. A., Macedo de Sousa, B., & Bravo, M. (2020). Use of Virtual Reality for the Management of Anxiety and Pain in Dental Treatments: Systematic Review and Meta-Analysis*. Journal of Clinical Medicine, 9(4),* 1025.

Maciołek, J., Wąsek, W., Kamiński, B., Piotrowicz, K., & Krzesiński, P. (2020). The impact of mobile virtual reality–enhanced relaxation training on anxiety levels in patients undergoing cardiac rehabilitation*. Kardiologia Polska, 78(10),* 1032-1034.

McSherry, T., Atterbury, M., Gartner, S., Helmold, E., Searles, D. M., & Schulman, C. (2018). Randomized, crossover study of immersive virtual reality to decrease opioid use during painful wound care procedures in adults. *Journal of Burn Care & Research, 39(2),* 278-285.

Mitrakul, K., Asvanund, Y., Arunakul, M., & Paka-Akekaphat, S. (2015). Effect of audiovisual eyeglasses during dental treatment in 5-8 year-old children Introduction. *European Journal of Paediatric Dentistry, 16(239),* 26418930.

Mohammad, E. B., & Ahmad, M. (2019). Virtual reality as a distraction technique for pain and anxiety among patients with breast cancer: A randomized control trial. *Palliative & Supportive Care, 17(1),* 29-34.

Momenyan, N., Safaei, A. A., & Hantoushzadeh, S. (2021). Immersive virtual reality analgesia in un-medicated laboring women (during stage 1 and 2): a randomized controlled trial. *Clinical and Experimental Obstetrics & Gynecology, 48(1),* 110-116.

Morgan, H., Nana, M., Phillips, D., & Gallagher, S. (2021). The Effect of a VIrtual RealiTy Immersive Experience Upon Anxiety Levels, Procedural Understanding, and Satisfaction in Patients Undergoing CArdiac CaTHeterization: The VIRTUAL CATH Trial. *The Journal of Invasive Cardiology, 33(9),* e681-e686.

Morris, L. D., Louw, Q. A., & Crous, L. C. (2010). Feasibility and potential effect of a low-cost virtual reality system on reducing pain and anxiety in adult burn injury patients during physiotherapy in a developing country. *Burns, 36(5),* 659-664.

Niharika, P., Reddy, N. V., Srujana, P., Srikanth, K., Daneswari, V., & Geetha, K. S. (2018). Effects of distraction using virtual reality technology on pain perception and anxiety levels in children during pulp therapy of primary molars. *Journal of Indian Society of Pedodontics and Preventive Dentistry, 36(4),* 364-369.

Noben, L., Goossens, S. M. T. A., Truijens, S. E. M., Van Berckel, M. M. G., Perquin, C. W., Slooter, G. D., & Van Rooijen, S. J. (2019). A virtual reality video to improve information provision and reduce anxiety before cesarean delivery: randomized controlled trial. *JMIR mental health, 6(12),* e15872.

Nunna, M., Dasaraju, R. K., Kamatham, R., Mallineni, S. K., & Nuvvula, S. (2019). Comparative evaluation of virtual reality distraction and counter-stimulation on dental anxiety and pain perception in children. *Journal of Dental Anesthesia and Pain Medicine, 19(5),* 277.

Obrero-Gaitán, E., Cortés-Pérez, I., Calet-Fernández, T., García-López, H., López Ruiz, M. d. C., & Osuna-Pérez, M. C. (2022). Digital and Interactive Health Interventions Minimize the Physical and Psychological Impact of Breast Cancer, Increasing Women’s Quality of Life: A Systematic Review and Meta-Analysis. *Cancers, 14(17),* 4133.

Özkan, T. K., & Polat, F. (2020). The effect of virtual reality and kaleidoscope on pain and anxiety levels during venipuncture in children. Journal of *PeriAnesthesia Nursing, 35(2),* 206-211.

Pande, P., Rana, V., Srivastava, N., & Kaushik, N. (2020). Effectiveness of different behavior guidance techniques in managing children with negative behavior in a dental setting: A randomized control study. *Journal of Indian Society of Pedodontics and Preventive Dentistry, 38(3),* 259-265.

Park, J., Nahm, F., Kim, J., Jeon, Y., Ryu, J., & Han, S. (2019). The effect of mirroring display of virtual reality tour of the operating theatre on preoperative anxiety: a randomized controlled trial. *IEEE Journal of Biomedical and Health Informatics, 23(6),* 2655-2660.

Piskorz, J., & Czub, M. (2018). Effectiveness of a virtual reality intervention to minimize pediatric stress and pain intensity during venipuncture. *Journal for Specialists in Pediatric Nursing, 23(1),* e12201.

Ran, L., Zhao, N., Fan, L., Zhou, P., Zhang, C., & Yu, C. (2021). Application of virtual reality on non-drug behavioral management of short-term dental procedure in children. *Trials, 22,* 1-9.

Robertson, A., Khan, R., Fick, D., Robertson, W.B., Gunaratne, D.R., Yapa, S., Bowden, V., Hoffman, H. and Rajan, R., 2017, June. The effect of Virtual Reality in reducing preoperative anxiety in patients prior to arthroscopic knee surgery: A randomised controlled trial. In *5th IEEE International Conference on Serious Games and Applications for Health*, SeGAH 2017. IEEE, Institute of Electrical and Electronics Engineers.

Ryu, J., Park, J., Nahm, F., Jeon, Y., Oh, A., Lee, H., Kim, J., & Han, S. (2018). The effect of gamification through a virtual reality on preoperative anxiety in pediatric patients undergoing general anesthesia: a prospective, randomized, and controlled trial. *Journal of Clinical Medicine, 7(9),* 284.

Ryu, J., Park, S., Park, J., Kim, J., Yoo, H., Kim, T., Hong, J., & Han, S. (2017). Randomized clinical trial of immersive virtual reality tour of the operating theatre in children before anaesthesia. *Journal of British Surgery, 104(12),* 1628-1633.

Ryu, J., Oh, A., Yoo, H., Kim, J., Park, J., & Han, S. (2019). The effect of an immersive virtual reality tour of the operating theater on emergence delirium in children undergoing general anesthesia: A randomized controlled trial. *Pediatric Anesthesia, 29(1),* 98-105.

Schneider, S. M., & Workman, M. (1999). Effects of virtual reality on symptom distress in children receiving chemotherapy. *CyberPsychology & Behavior, 2(2),* 125-134.

Sharifpour, S., Manshaee, G. R., & Sajjadian, I. (2021). Effects of virtual reality therapy on perceived pain intensity, anxiety, catastrophising and self‐efficacy among adolescents with cancer. *Counselling and Psychotherapy Research, 21(1),* 218-226.

Shetty, V., Suresh, L. R., & Hegde, A. M. (2019). Effect of virtual reality distraction on pain and anxiety during dental treatment in 5 to 8 year old children. *Journal of Clinical Pediatric Dentistry, 43(2),* 97-102.

Simonetti, V., Tomietto, M., Comparcini, D., Vankova, N., Marcelli, S., & Cicolini, G. (2022). Effectiveness of virtual reality in the management of paediatric anxiety during the peri‑operative period: A systematic review and meta-analysis. *International Journal of Nursing Studies, 125,* 104115.

Szczepańska-Gieracha, J., Jóźwik, S., Cieślik, B., Mazurek, J., & Gajda, R. (2021). Immersive virtual reality therapy as a support for cardiac rehabilitation: a pilot randomized-controlled trial. *Cyberpsychology, Behavior, and Social Networking, 24(8),* 543-549.

Tas, F. Q., van Eijk, C. A. M., Staals, L. M., Legerstee, J. S., & Dierckx, B. (2022). Virtual reality in pediatrics, effects on pain and anxiety: A systematic review and meta-analysis update. *Pediatric Anesthesia, 32(12),* 1292-1304.

Tejera, M. D., Beltran-Alacreu, H., Cano-de-la-Cuerda, R., Leon Hernandez, J. V., Martín-Pintado-Zugasti, A., Calvo-Lobo, C., Gil-Martínez, A., & Fernández-Carnero, J. (2020). Effects of virtual reality versus exercise on pain, functional, somatosensory and psychosocial outcomes in patients with non-specific chronic neck pain: a randomized clinical trial*. International Journal of Environmental Research and Public Health, 17(16),* 5950.

Tennant, M., Youssef, G. J., McGillivray, J., Clark, T-J., McMillan, L., & McCarthy, M. C. (2020). Exploring the use of immersive virtual reality to enhance psychological well-being in pediatric oncology: a pilot randomized controlled trial. *European Journal of Oncology Nursing, 48,* 101804.

Turan Kavradim, S., Yangöz, Ş. T., & Özer, Z. (2023). Effectiveness of virtual reality interventions on physiological and psychological outcomes of adults with cardiovascular disease: A systematic review and meta-analysis. *Journal of Nursing Scholarship, 55(5),* 949-966.

Turrado, V., Guzmán, Y., Jiménez-Lillo, J., Villegas, E., de Lacy, F. B., Blanch, J., Balibrea, J. M., & Lacy, A. (2021). Exposure to virtual reality as a tool to reduce peri-operative anxiety in patients undergoing colorectal cancer surgery: a single-center prospective randomized clinical trial. *Surgical endoscopy, 35,* 4042-4047.

van Twillert, B., Bremer, M., & Faber, A. W. (2007). Computer-generated virtual reality to control pain and anxiety in pediatric and adult burn patients during wound dressing changes. *Journal of Burn Care & Research, 28(5),* 694-702.

Wang, Y., Guo, L., & Xiong, X. (2022). Effects of Virtual Reality-Based Distraction of Pain, Fear, and Anxiety During Needle-Related Procedures in Children and Adolescents. *Frontiers in Psychology,* 13.

Wolitzky, K., Fivush, R., Zimand, E., Hodges, L., & Rothbaum, B. O. (2005). Effectiveness of virtual reality distraction during a painful medical procedure in pediatric oncology patients. *Psychology and Health, 20(6),* 817-824.

Wong, C. L., Li, C. K., Chan, C. W., Choi, K. C., Chen, J., Yeung, M. T., & Chan, O. N. (2020). Virtual reality intervention targeting pain and anxiety among pediatric cancer patients undergoing peripheral intravenous cannulation: a randomized controlled trial. *Cancer Nursing, 44(6),* 435-442.

Wu, Y., Wang, N., Zhang, H., Sun, X., Wang, Y., & Zhang, Y. (2023). Effectiveness of Virtual Reality in Symptom Management of Cancer Patients: A Systematic Review and Meta-Analysis. *Journal of Pain and Symptom Management, 65(5),* e467-e482.

Xu, N., Chen, S., Liu, Y., Jing, Y., & Gu, P. (2022). The Effects of Virtual Reality in Maternal Delivery: Systematic Review and Meta-analysis. *JMIR Serious Games, 10(4),* e36695.

Yan, X., Yan, Y., Cao, M., Xie, W., O'Connor, S., Lee, J. J., & Ho, M-H. (2023). Effectiveness of virtual reality distraction interventions to reduce dental anxiety in paediatric patients: A systematic review and meta-analysis. Journal of Dentistry, 132, 104455.

Yang, J., Ryu, J., Nam, E., Lee, H., & Lee, J. (2019). Effects of preoperative virtual reality magnetic resonance imaging on preoperative anxiety in patients undergoing arthroscopic knee surgery: a randomized controlled study. *Arthroscopy: The Journal of Arthroscopic & Related Surgery, 35(8),* 2394-2399.

Zeng, Y., Zhang, J-E., Cheng, A., Cheng, H., & Wefel, J. (2019). Meta-Analysis of the Efficacy of Virtual Reality–Based Interventions in Cancer-Related Symptom Management. Integrative Cancer Therapies, 18, 1534735419871108.

Zhang, H., Xu, H., Zhang, Z-X., & Zhang, Q. (2022). Efficacy of virtual reality-based interventions for patients with breast cancer symptom and rehabilitation management: a systematic review and meta-analysis. *BMJ Open, 12(3),* e051808.
