# Supplementary material for: Extended Reality Interventions for Health and Procedural Anxiety: Panoramic Meta-Analysis Based on Overviews of Reviews
Source: J Med Internet Res. 2025 Jan 8;27:e58086. doi: 10.2196/58086 (PMC11754977; doi:10.2196/58086)
Supplement: Multimedia Appendix 4 [file jmir_v27i1e58086_app4.docx]

**Multimedia Appendix 4.**  Appraisals of Risk of Bias (RoB) and Methodological Quality

| **Parent Review** | **Study** | **Appraisal Method** | **Extracted Data** |
| --- | --- | --- | --- |
| Kılıç *et al.* (2021) | Gujjar *et al.* (2019) | Cochrane RoB and ROBINS-I tools | RoB appraised as low for three criteria, but unknown for three items. For our analyses, overall RoB is therefore evaluated as unknown. |
| Kılıç *et al.* (2021) | Van Twillert *et al.* (2007) | Cochrane RoB and ROBINS-I tools | RoB appraised as high for performance and detection bias and unknown for the five remaining criteria. For our analyses, overall RoB is therefore evaluated as high. |
| Kılıç *et al.* (2021) | McSherry *et al.* (2018) | Cochrane RoB and ROBINS-I tools | RoB appraised as high for ‘other risk’ item and unknown for the five remaining criteria. For our analyses, overall RoB is therefore evaluated as high. |
| Kılıç *et al.* (2021) | Lahti *et al.* (2020) | Cochrane RoB and ROBINS-I tools | RoB appraised as low for one criterion, but high for one (selection bias) and unknown for four items. For our analyses, overall RoB is therefore evaluated as high. |
| Kılıç *et al.* (2021) | Gold & Mahrer (2018) | Cochrane RoB and ROBINS-I tools | RoB appraised as low for two criteria, but high for one (performance and detection bias) and unknown for three items. For our analyses, overall RoB is therefore evaluated as high. |
| Kılıç *et al.* (2021) | Jiang *et al.* (2020) | Cochrane RoB and ROBINS-I tools | RoB appraised as low for four criteria, but high for performance and detection bias and unknown for ‘other bias’ item. For our analyses, overall RoB is therefore evaluated as high. |
| Kılıç *et al.* (2021) | Nunna *et al.* (2019) | Cochrane RoB and ROBINS-I tools | RoB appraised as unknown for all evaluated criteria. |
| Kılıç *et al.* (2021) | Niharika *et al.* (2018) | Cochrane RoB and ROBINS-I tools | RoB appraised as high for performance and detection bias and unknown for the five remaining criteria. Overall RoB is therefore high. |
| Kılıç *et al.* (2021) | Gerçeker- *et al.* (2020) | Cochrane RoB and ROBINS-I tools | RoB appraised as low for five criteria, but unknown for the ‘other bias’ item. Overall RoB is therefore unknown. |
| Kılıç *et al.* (2021) | Aminabadi *et al.* (2012) | Cochrane RoB and ROBINS-I tools | RoB appraised as low for one criterion, but high for two (selection and other bias) and unknown for three items. Overall RoB is therefore high. |
| Koo *et al.* (2020) | Bekelis *et al.* (2017) | Cochrane RoB tool | RoB appraised as low for all six criteria. |
| Koo *et al.* (2020) | Haisely *et al.* (2020) | Cochrane RoB tool | RoB appraised as low for four criteria, but unknown for two others. For our analyses, overall RoB is therefore evaluated as unknown. |
| Koo *et al.* (2020) | Noben *et al.* (2019) | Cochrane RoB tool | RoB appraised as low for four criteria, but unknown for two others. For our analyses, overall RoB is therefore evaluated as unknown. |
| Koo *et al.* (2020) | Robertson *et al.* (2017) | Cochrane RoB tool | RoB appraised as low for all criteria. |
| Koo *et al.* (2020) | Yang *et al.* (2019) | Cochrane RoB tool | RoB appraised as low for five criteria, but unknown for selection bias. For our analyses, overall RoB is therefore evaluated as unknown. |
| Koo *et al.* (2020) | Dehghan *et al.* (2019) | Cochrane RoB tool | RoB appraised as low for four criteria, but unknown for two others. For our analyses, overall RoB is therefore evaluated as unknown. |
| Koo *et al.* (2020) | Eijlers *et al.* (2019) | Cochrane RoB tool | RoB appraised as low for five criteria, but unknown for selection bias. For our analyses, overall RoB is therefore evaluated as unknown. |
| Koo *et al.* (2020) | Ryu *et al.* (2017) | Cochrane RoB tool | RoB appraised as low for all evaluated criteria. |
| Koo *et al.* (2020) | Ryu *et al.* (2018) | Cochrane RoB tool | RoB appraised as low for all evaluated criteria. |
| Koo *et al.* (2020) | Ryu *et al.* (2019) | Cochrane RoB tool | RoB appraised as low for all evaluated criteria. |
| Tas *et al.* (2022) | Chan *et al.* (2019a) | Delphi list | RoB not specifically appraised. Methodological quality rating provided a score of 6/6. |
| Tas *et al.* (2022) | Chan *et al.* (2019b) | Delphi list | RoB not specifically appraised. Methodological quality rating provided a score of 6/6. |
| Tas *et al.* (2022) | Dumoulin *et al.* (2019) | Delphi list | RoB not specifically appraised. Methodological quality rating provided a score of 6/6. |
| Tas *et al.* (2022) | Jung *et al.* (2020) | Delphi list | RoB not specifically appraised. Methodological quality rating provided a score of 6/6. |
| Tas *et al.* (2022) | Liu *et al.* (2020) | Delphi list | RoB not specifically appraised. Methodological quality rating provided a score of 5/6. |
| Tas *et al.* (2022) | Schneider *et al.* (1999) | Delphi list | RoB not specifically appraised. Methodological quality rating provided a score of 2/6. |
| Tas *et al.* (2022) | Gershon *et al.* (2004) | Delphi list | RoB not specifically appraised. Methodological quality rating provided a score of 4/6. |
| Tas *et al.* (2022) | Wolitzky *et al.* (2005) | Delphi list | RoB not specifically appraised. Methodological quality rating provided a score of 4/6. |
| Tas *et al.* (2022) | Piskorz & Czub (2017) | Delphi list | RoB not specifically appraised. Methodological quality rating provided a score of 1/6. |
| Tas *et al.* (2022) | Han *et al.* (2019) | Delphi list | RoB not specifically appraised. Methodological quality rating provided a score of 7/8. |
| Simonetti *et al.* (2022) | Park *et al.* (2019) | Cochrane RoB tool | RoB appraised as low for all evaluated criteria. |
| Yan *et al.* (2023) | Aditya *et al.* (2021) | Cochrane RoB-2 tool | Overall RoB: ‘Some Concerns’. Concerns highlighted regarding deviations from the intended interventions and in selection of the reported result. |
| Yan *et al.* (2023) | Aminabadi *et al.* (2022) | Cochrane RoB-2 tool | Overall RoB: ‘Some Concerns’. Concerns highlighted regarding deviations from the intended interventions and in selection of the reported result. |
| Yan *et al.* (2023) | Buldur & Candan (2021) | Cochrane RoB-2 tool | Overall RoB: ‘Some Concerns’. Concerns highlighted regarding deviations from the intended interventions and in selection of the reported result. |
| Yan *et al.* (2023) | Du *et al.* (2022) | Cochrane RoB-2 tool | Overall RoB: ‘High’. Risks related to the measurement of the outcome, while concerns regarded deviations from the intended interventions and in selection of the reported result. |
| Yan *et al.* (2023) | Gomez-Polo *et al.* (2021) | Cochrane RoB-2 tool | Overall RoB: ‘Some Concerns’. Concerns highlighted regarding deviations from the intended interventions and in selection of the reported result. |
| Yan *et al.* (2023) | Greeshma *et al.* (2021) | Cochrane RoB-2 tool | Overall RoB: ‘Some Concerns’. Concerns highlighted regarding the randomisation process, deviations from the intended interventions, and in selection of the reported result. |
| Yan *et al.* (2023) | Pande *et al.* (2020) | Cochrane RoB-2 tool | Overall RoB: ‘Some Concerns’. Concerns highlighted regarding deviations from the intended interventions and in selection of the reported result. |
| Yan *et al.* (2023) | Ran *et al.* (2020) | Cochrane RoB-2 tool | Overall RoB: ‘High’. Risks related to deviations from the intended interventions, while concerns regarded in selection of the reported result. |
| Yan *et al.* (2023) | Shetty *et al.* (2019) | Cochrane RoB-2 tool | Overall RoB: ‘Some Concerns’. Concerns highlighted regarding the randomisation process, deviations from the intended interventions, and in selection of the reported result. |
| Lopez-Valverde *et al.* (2023) | Mitrakul *et al.* (2015) | Cochrane RoB tool | Not specified within review article. |
| Lopez-Valverde *et al.* (2023) | Asvanund *et al.* (2015) | Cochrane RoB tool | Not specified within review article. |
| Lopez-Valverde *et al.* (2023) | Al-Khotani *et al.* (2016) | Cochrane RoB tool | Not specified within review article. |
| Gao *et al.* (2023) | Canares *et al.* (2021) | Cochrane RoB tool | RoB appraised as low for four criteria, but high for two (performance bias and detection bias) and unknown for selection bias. For our analyses, overall RoB is therefore evaluated as high. |
| Gao *et al.* (2023) | Gold *et al.* (2021) | Cochrane RoB tool | RoB appraised as low for four criteria, but high for two (performance bias and detection bias) and unknown for selection bias. For our analyses, overall RoB is therefore evaluated as high. |
| Gao *et al.* (2023) | Goldman *et al.* (2021a) | Cochrane RoB tool | RoB appraised as low for five criteria, but unknown for two criteria. For our analyses, overall RoB is therefore evaluated as unknown. |
| Gao *et al.* (2023) | Goldman *et al.* (2021b) | Cochrane RoB tool | RoB appraised as low for five criteria, but unknown for two criteria. For our analyses, overall RoB is therefore evaluated as unknown. |
| Gao *et al.* (2023) | Hundert *et al.* (2021) | Cochrane RoB tool | RoB appraised as low for four criteria, but high for two (performance bias and detection bias) and unknown for selection bias. For our analyses, overall RoB is therefore evaluated as high. |
| Gao *et al.* (2023) | İnangil *et al.* (2020) | Cochrane RoB tool | RoB appraised as low for five criteria, but unknown for two criteria. For our analyses, overall RoB is therefore evaluated as unknown. |
| Gao *et al.* (2023) | Litwin *et al.* (2021) | Cochrane RoB tool | RoB appraised as low for four criteria, but high for two (performance bias and detection bias) and unknown for selection bias. For our analyses, overall RoB is therefore evaluated as high. |
| Gao *et al.* (2023) | Gerçeker- *et al.* (2021) | Cochrane RoB tool | RoB appraised as low for four criteria, but high for two (performance bias and detection bias) and unknown for selection bias. For our analyses, overall RoB is therefore evaluated as high. |
| Wang *et al.* (2022) | Koc Ozkan & Polat (2020) | Cochrane RoB-2 tool | Overall RoB: ‘Low’. Unclear risk regarding deviations from intended interventions. |
| Turan-Kavradim *et al.* (2023) | Hessabi *et al.* (2020) | Cochrane RoB-2 tool | Overall RoB: ‘High’. Risks related to deviations from the intended interventions, while concerns regarded the randomisation process. |
| Turan-Kavradim *et al.* (2023) | Jóźwik *et al.* (2021a) | Cochrane RoB-2 tool | Overall RoB: ‘High’. Risks related to missing outcome data, while concerns regarded deviations from the intended interventions and in selection of the reported result. |
| Turan-Kavradim *et al.* (2023) | Jóźwik *et al.* (2021b) | Cochrane RoB-2 tool | Overall RoB: ‘High’. Risks related to missing outcome data, while concerns regarded deviations from the intended interventions and in selection of the reported result. |
| Turan-Kavradim *et al.* (2023) | Keshvari *et al.* (2021) | Cochrane RoB-2 tool | RoB appraised as low for all assessed criteria. |
| Turan-Kavradim *et al.* (2023) | Maciolek *et al.* (2020) | Cochrane RoB-2 tool | Overall RoB: ‘Some Concerns’. Concerns highlighted regarding the randomisation process. |
| Turan-Kavradim *et al.* (2023) | Morgan *et al.* (2021) | Cochrane RoB-2 tool | Overall RoB: ‘Some Concerns’. Concerns highlighted regarding the randomisation process and deviations from the intended interventions. |
| Turan-Kavradim *et al.* (2023) | Szczepańska-Gieracha *et al.* (2021) | Cochrane RoB-2 tool | Overall RoB: ‘Some Concerns’. Concerns highlighted regarding the randomisation process. |
| Lan *et al.* (2023) | Hoffman *et al.* (2000) | Cochrane RoB tool | RoB appraised as low for three criteria, but high for detection bias and unknown for three criteria. For our analyses, overall RoB is therefore evaluated as high. |
| Lan *et al.* (2023) | Hoffman *et al.* (2001) | Cochrane RoB tool | RoB appraised as low for two criteria, but high for two (detection bias and missing outcome data) and unknown for three criteria. For our analyses, overall RoB is evaluated as high. |
| Lan *et al.* (2023) | Morris *et al.* (2010) | Cochrane RoB tool | RoB appraised as low for five criteria, but high for detection bias and unknown for allocation concealment. For our analyses, overall RoB is therefore evaluated as high. |
| Gava *et al.* (2022) | Tejera *et al.* (2020) | PEDRO scale | RoB not specifically appraised. Methodological quality rated as ‘high’ overall. |
| Xu *et al.* (2022) | Akin *et al.* (2021) | Cochrane RoB-2 tool | Overall RoB: ‘Some Concerns’. High risks identified for deviations from the intended interventions, while there were concerns regarding missing outcome data. |
| Xu *et al.* (2022) | Li *et al.* (2020) | Cochrane RoB-2 tool | Overall RoB: ‘Some Concerns’. High risks identified for deviations from the intended interventions, while there were concerns regarding the randomisation process, missing outcome data, and in selection of the reported result. |
| Xu *et al.* (2022) | Lin *et al.* (2021) | Cochrane RoB-2 tool | Overall RoB: ‘Some Concerns’. High risks identified for deviations from the intended interventions, while there were concerns regarding the randomisation process, missing outcome data, and in selection of the reported result. |
| Xu *et al.* (2022) | Liu & Wan (2020) | Cochrane RoB-2 tool | Overall RoB: ‘Some Concerns’. High risks identified for deviations from the intended interventions, while there were concerns regarding the randomisation process, missing outcome data, and in selection of the reported result. |
| Xu *et al.* (2022) | Momenyan *et al.* (2021) | Cochrane RoB-2 tool | Overall RoB: ‘Some Concerns’. High risks identified for deviations from the intended interventions, while there were concerns regarding missing outcome data. |
| Baradwan *et al.* (2022) | Frey *et al.* (2018) | Cochrane RoB tool | RoB appraised as low for five criteria, but high for performance and detection bias items. For our analyses, overall RoB is therefore evaluated as high. |
| Wu *et al.* (2023) | Gao *et al.* (2020) | Cochrane RoB tool | RoB appraised as low for two criteria, but high for performance bias and unknown for four criteria. For our analyses, overall RoB is therefore evaluated as high. |
| Wu *et al.* (2023) | Mohammad & Ahmad (2019) | Cochrane RoB tool | RoB appraised as low for three criteria, but high for performance bias and unknown for three criteria. For our analyses, overall RoB is therefore evaluated as high. |
| Wu *et al.* (2023) | Tennant *et al.* (2020) | Cochrane RoB tool | RoB appraised as low for two criteria, but high for performance bias and unknown for four criteria. For our analyses, overall RoB is therefore evaluated as high. |
| Wu *et al.* (2023) | Turrado *et al.* (2021) | Cochrane RoB tool | RoB appraised as low for three criteria, but high for performance bias and unknown for three criteria. For our analyses, overall RoB is therefore evaluated as high. |
| Wu *et al.* (2023) | Wong *et al.* (2020) | Cochrane RoB tool | RoB appraised as low for four criteria, but high for performance bias and unknown for two criteria. For our analyses, overall RoB is therefore evaluated as high. |
| Wu *et al.* (2023) | Zhang *et al.* (2022) | Cochrane RoB tool | RoB appraised as low for five criteria, but high for performance bias and unknown for the ‘other bias’ item. For our analyses, overall RoB is therefore evaluated as high. |
| Obrero-Gaitan *et al.* (2022) | Chirico *et al.* (2019) | Cochrane RoB tool | RoB appraised as low for two criteria, but high for three (selection, performance, and detection bias) and unknown for the ‘other bias’ item. For our analyses, overall RoB is therefore evaluated as high. |
| Obrero-Gaitan *et al.* (2022) | Jimenez *et al.* (2018) | Cochrane RoB tool | RoB appraised as low for two criteria, but high for three (selection, performance, and detection bias) and unknown for the ‘other bias’ item. For our analyses, overall RoB is therefore evaluated as high. |
| Czech *et al.* (2023) | Sharifpour *et al.* (2021) | Cochrane RoB-2 tool | Overall RoB: ‘Some Concerns’. Concerns highlighted regarding the randomisation process, deviations from the intended interventions, measurement of the outcome, and in selection of the reported result. |
| Bu *et al.* (2022) | Buche *et al.* (2021^a^) | Cochrane RoB and ROBINS-I tools | Overall RoB: ‘Moderate Risk’. Risks highlighted regarding biases due to confounding factors, deviations from intended interventions, missing outcome data, measurement of the outcome, and in selection of the reported result. |
| Bu *et al.* (2022) | Buche *et al.* (2021^b^) | Cochrane RoB and ROBINS-I tools | Overall RoB: ‘Moderate Risk’. Risks highlighted regarding biases due to confounding factors, deviations from intended interventions, missing outcome data, measurement of the outcome, and in selection of the reported result. |
| Zeng *et al.* (2019) | Baños *et al.* (2013) | 8-item Quality and RoB assessment tool | Overall RoB: ‘High Risk’. Risks highlighted regarding study randomisation, control, missing data, power analysis, and follow-up methods. |
| Zeng *et al.* (2019) | Glennon *et al.* (2018) | 8-item Quality and RoB assessment tool | Overall RoB: ‘High Risk’. Risks highlighted regarding study randomisation, missing data, power analysis, and follow-up methods. |

References

Aditya, P., Prasad, M. G., Nagaradhakrishna, A., Raju, N. S., & Babu, D. N. (2021). Comparison of effectiveness of three distraction techniques to allay dental anxiety during inferior alveolar nerve block in children: A randomized controlled clinical trial. *Heliyon, 7(9)*.

Al-Khotani, A., Bello, L. A. a., & Christidis, N. (2016). Effects of audiovisual distraction on children’s behaviour during dental treatment: a randomized controlled clinical trial. *Acta Odontologica Scandinavica, 74(6),* 494-501.

Aminabadi, N. A., Erfanparast, L., Sohrabi, A., Oskouei, S. G., & Naghili, A. (2012). The impact of virtual reality distraction on pain and anxiety during dental treatment in 4-6 year-old children: a randomized controlled clinical trial. *Journal of Dental Research, Dental Clinics, Dental Prospects, 6(4),* 117.

Aminabadi, N. A., Golsanamlou, O., Halimi, Z., & Jamali, Z. (2022). Assessing the different levels of virtual reality that influence anxiety, behavior, and oral health status in preschool children: Randomized controlled clinical trial. *JMIR Perioperative Medicine, 5(1),* e35415.

Asvanund, Y., Mitrakul, K., Juhong, R-O., & Arunakul, M. (2015). Effect of audiovisual eyeglasses during local anesthesia injections in 5-to 8-year-old children. *Quintessence International, 46(6)*.

Baños, R., Espinoza, M., García-Palacios, A., Cervera, J. M., Esquerdo, G., Barrajón, E., & Botella, C. (2013). A positive psychological intervention using virtual reality for patients with advanced cancer in a hospital setting: a pilot study to assess feasibility. *Supportive Care in Cancer, 21,* 263-270.

Baradwan, S., Khadawardi, K., Badghish, E., Alkhamis, W., Dahi, A., Abdallah, K., Kamel, M., Sayd, Z., Mohamed, M., Ali, H., Elhalim, A., Mahmoud, M., Mohamed, A., Mohamed, D., Shama, A., Hagras, A., Ali, H., Abdelhakim, A., Saleh, M., . . . Bakry, M. (2022). The impact of virtual reality on pain management during normal labor: A systematic review and <i>meta</i>-analysis of randomized controlled trials. *Sexual & Reproductive Healthcare, 32,* 100720.

Bekelis, K., Calnan, D., Simmons, N., MacKenzie, T., & Kakoulides, G. (2017). Effect of an Immersive Preoperative Virtual Reality Experience on Patient Reported Outcomes: *A Randomized Controlled Trial. Annals of Surgery, 265(6),* 1068-1073.

Bu, X., Ng, P. H. F., Xu, W., Cheng, Q., Chen, P. Q., Cheng, A. S. K., & Liu, X. (2022). The Effectiveness of Virtual Reality–Based Interventions in Rehabilitation Management of Breast Cancer Survivors: Systematic Review and Meta-analysis. *JMIR Serious Games, 10(1),* e31395.

Buche, H., Michel, A., Piccoli, C., & Blanc, N. (2021). Contemplating or acting? Which immersive modes should be favored in virtual reality during physiotherapy for breast cancer rehabilitation. *Frontiers in Psychology, 12*, 631186.

Buldur, B., & Candan, M. (2020). Does virtual reality affect children’s dental anxiety, pain, and behaviour? a randomised, placebo-controlled, cross-over trial. *Pesquisa Brasileira em Odontopediatria e Clínica Integrada, 21*, e0082.

Canares, T., Parrish, C., Santos, C., Badawi, A., Stewart, A., Kleinman, K., Psoter, K., & McGuire, J. (2021). Pediatric coping during venipuncture with virtual reality: Pilot randomized controlled trial. *JMIR Pediatrics and Parenting, 4(3),* e26040.

Chan, E., Hovenden, M., Ramage, E., Ling, N., Pham, J. H., Rahim, A., Lam, C., Liu, L., Foster, S., & Sambell, R. (2019). Virtual reality for pediatric needle procedural pain: two randomized clinical trials. *The Journal of Pediatrics, 209,* 160-167.

Chirico, A., Maiorano, P., Indovina, P., Milanese, C., Giordano, G. G., Alivernini, F., Iodice, G., Gallo, L., De Pietro, G., & Lucidi, F. (2020). Virtual reality and music therapy as distraction interventions to alleviate anxiety and improve mood states in breast cancer patients during chemotherapy. *Journal of Cellular Physiology, 235(6),* 5353-5362.

Czech, O., Rutkowski, S., Kowaluk, A., Kiper, P., & Malicka, I. (2023). Virtual reality in chemotherapy support for the treatment of physical functions, fear, and quality of life in pediatric cancer patients: A systematic review and meta-analysis. *Frontiers in Public Health, 11*.

Dehghan, F., Jalali, R., & Bashiri, H. (2019). The effect of virtual reality technology on preoperative anxiety in children: a Solomon four-group randomized clinical trial. *Perioperative Medicine, 8,* 1-7.

Du, Q., Ma, X., Wang, S., Zhou, S., Luo, C., Tian, K., Fei, W., & Liu, X. (2022). A digital intervention using virtual reality helmets to reduce dental anxiety of children under local anesthesia and primary teeth extraction: A randomized clinical trial. *Brain and Behavior, 12(6),* e2600.

Dumoulin, S., Bouchard, S., Ellis, J., Lavoie, K. L., Vézina, M-P., Charbonneau, P., Tardif, J., & Hajjar, A. (2019). A randomized controlled trial on the use of virtual reality for needle-related procedures in children and adolescents in the emergency department. *Games for Health, 8(4),* 285-293.

Eijlers, R., Utens, E. M. W. J., Staals, L. M., de Nijs, P. F. A., Berghmans, J. M., Wijnen, R. M. H., Hillegers, M. H. J., Dierckx, B., & Legerstee, J. S. (2019). Systematic Review and Meta-analysis of Virtual Reality in Pediatrics: Effects on Pain and Anxiety. *Anesthesia and Analgesia, 129(5),* 1344-1353.

Frey, D. P., Bauer, M. E., Bell, C. L., Low, L. K., Hassett, A. L., Cassidy, R. B., Boyer, K. D., & Sharar, S. R. (2019). Virtual reality analgesia in labor: the VRAIL pilot study—a preliminary randomized controlled trial suggesting benefit of immersive virtual reality analgesia in unmedicated laboring women. *Anesthesia & Analgesia, 128(6),* e93-e96.

Gao, J., Liu, S., Zhang, S., Wang, Y., Liang, Z., Feng, Q., Hu, M., & Zhang, Q. (2022). Pilot study of a virtual reality educational intervention for radiotherapy patients prior to initiating treatment. *Journal of Cancer Education,* 1-8.

Gao, Y., Xu, Y., Liu, N., & Fan, L. (2023). Effectiveness of virtual reality intervention on reducing the pain, anxiety and fear of needle‐related procedures in paediatric patients: A systematic review and meta‐analysis. *Journal of Advanced Nursing, 79(1),* 15-30.

Gava, V., Fialho, H., Calixtre, L., Barbosa, G., & Kamonseki, D. (2022). Effects of Gaming on Pain-Related Fear, Pain Catastrophizing, Anxiety, and Depression in Patients with Chronic Musculoskeletal Pain: A Systematic Review and Meta-Analysis. *Games for Health*, *11(6),* 369-384.

Gerçeker, G. Ö., Ayar, D., Özdemir, E. Z., & Bektaş, M. (2020). Effects of virtual reality on pain, fear and anxiety during blood draw in children aged 5–12 years old: A randomised controlled study. *Journal of Clinical Nursing, 29(7-8),* 1151-1161.

Gerçeker, G. Ö., Bektaş, M., Aydınok, Y., Ören, H., Ellidokuz, H., & Olgun, N. (2021). The effect of virtual reality on pain, fear, and anxiety during access of a port with huber needle in pediatric hematology-oncology patients: Randomized controlled trial. *European Journal of Oncology Nursing, 50,* 101886.

Gershon, J., Zimand, E., Pickering, M., Rothbaum, B. O., & Hodges, L. (2004). A pilot and feasibility study of virtual reality as a distraction for children with cancer. *Journal of the American Academy of Child & Adolescent Psychiatry, 43(10),* 1243-1249.

Glennon, C., McElroy, S. F., Connelly, L. M., Lawson, L. M., Bretches, A. M., Gard, A. R., & Newcomer, L. R. (2018). Use of Virtual Reality to Distract From Pain and Anxiety. In *Oncology Nursing Forum* (Vol. 45, No. 4).

Gold, J. I., & Mahrer, N. E. (2018). Is virtual reality ready for prime time in the medical space? A randomized control trial of pediatric virtual reality for acute procedural pain management*. Journal of Pediatric Psychology, 43(3),* 266-275.

Gold, J. I., SooHoo, M., Laikin, A. M., Lane, A. S., & Klein, M. J. (2021). Effect of an immersive virtual reality intervention on pain and anxiety associated with peripheral intravenous catheter placement in the pediatric setting: a randomized clinical trial. *JAMA Network Open, 4(8),* e2122569-e2122569.

Goldman, R. D., & Behboudi, A. (2021a). Virtual reality for intravenous placement in the emergency department—a randomized controlled trial. *European Journal of Pediatrics, 180,* 725-731.

Goldman, R. D., & Behboudi, A. (2021b). Pilot randomized controlled trial of virtual reality vs. standard-of-care during pediatric laceration repair. *Journal of Child & Adolescent Trauma, 14,* 295-298.

Gómez-Polo, C., Vilches, A., Ribas, D., Castaño-Séiquer, A., & Montero, J. (2021). Behaviour and anxiety management of paediatric dental patients through virtual reality: A randomised clinical trial. *Journal of Clinical Medicine, 10(14),* 3019.

Greeshma, G., George, S., Anandaraj, S., Sain, S., Jose, D., Sreenivas, A., Pillai, G., & Mol, N. (2021). Comparative evaluation of the efficacy of virtual reality distraction, audio distraction and tell-show-do techniques in reducing the anxiety level of pediatric dental patients: An in vivo study. *International Journal of Clinical Pediatric Dentistry,* 14(2), S173.

Gujjar, K. R., van Wijk, A., Kumar, R., & de Jongh, A. (2019). Efficacy of virtual reality exposure therapy for the treatment of dental phobia in adults: A randomized controlled trial. *Journal of Anxiety Disorders, 62,* 100-108.

Haisley, K. R., Straw, O. J., Müller, D. T., Antiporda, M. A., Zihni, A. M., Reavis, K. M., Bradley, D. D., & Dunst, C. M. (2020). Feasibility of implementing a virtual reality program as an adjuvant tool for peri-operative pain control; results of a randomized controlled trial in minimally invasive foregut surgery. *Complementary Therapies in Medicine, 49,* 102356.

Han, S., Park, J., Choi, S., Kim, J., Lee, H., Yoo, H., & Ryu, J. (2019). Effect of immersive virtual reality education before chest radiography on anxiety and distress among pediatric patients: a randomized clinical trial. *JAMA pediatrics, 173(11),* 1026-1031.

Hessabi, M., Sajjadi, M., Shareinia, H., & Rouhani, M. (2020). The effect of virtual reality on anxiety and quality of sleep in patients in cardiac care unit. *International Journal of Pharmaceutical and Phytopharmacological Research, 10*, 37-42.

Hoffman, H. G., Doctor, J. N., Patterson, D. R., Carrougher, G. J., & Furness, T. A. (2000). Virtual reality as an adjunctive pain control during burn wound care in adolescent patients. *Pain, 85,* 305-309.

Hoffman, H. G., Patterson, D. R., Carrougher, G. J., & Sharar, S. R. (2001). Effectiveness of virtual reality–based pain control with multiple treatments. *The Clinical journal of Pain, 17(3),* 229-235.

Hundert, A. S., Birnie, K. A., Abla, O., Positano, K., Cassiani, C., Lloyd, S., Tiessen, P. H., Lalloo, C., Jibb, L. A., & Stinson, J. (2022). A pilot randomized controlled trial of virtual reality distraction to reduce procedural pain during subcutaneous port access in children and adolescents with cancer. *The Clinical journal of Pain, 38(3),* 189-196.

İnangil, D., Şendir, M., & Büyükyılmaz, F. (2020). Efficacy of cartoon viewing devices during phlebotomy in children: a randomized controlled trial. *Journal of PeriAnesthesia Nursing, 35(4),* 407-412.

Jiang, M. Y. W., Upton, E., & Newby, J. M. (2020). A randomised wait-list controlled pilot trial of one-session virtual reality exposure therapy for blood-injection-injury phobias. *Journal of Affective Disorders, 276,* 636-645.

Jimenez, Y. A., Cumming, S., Wang, W., Stuart, K., Thwaites, D. I., & Lewis, S. J. (2018). Patient education using virtual reality increases knowledge and positive experience for breast cancer patients undergoing radiation therapy. *Supportive Care in Cancer, 26,* 2879-2888.

Jóźwik, S., Cieślik, B., Gajda, R., & Szczepańska-Gieracha, J. (2021a). Evaluation of the impact of virtual reality-enhanced cardiac rehabilitation on depressive and anxiety symptoms in patients with coronary artery disease: a randomised controlled trial. *Journal of Clinical Medicine, 10(10),* 2148.

Jóźwik, S., Cieślik, B., Gajda, R., & Szczepańska-Gieracha, J. (2021b). The use of virtual therapy in cardiac rehabilitation of female patients with heart disease. *Medicina, 57(8),* 768.

Jung, M. J., Libaw, J. S., Ma, K., Whitlock, E. L., Feiner, J. R., & Sinskey, J. L. (2020). Pediatric Distraction on Induction of Anesthesia With Virtual Reality and Perioperative Anxiolysis: A Randomized Controlled Trial. *Anesthesia & Analgesia, 132(3),* 798-806.

Keshvari, M., Yeganeh, M. R., Paryad, E., Roushan, Z. A., & Pouralizadeh, M. (2021). The effect of virtual reality distraction on reducing patients' anxiety before coronary angiography: a randomized clinical trial study. *The Egyptian Heart Journal, 73,* 1-8.

Kılıç, A., Brown, A., Aras, I., Hui, R., Hare, J., Hughes, L. D., & McCracken, L. M. (2021). Using Virtual Technology for Fear of Medical Procedures: A Systematic Review of the Effectiveness of Virtual Reality-Based Interventions. *Annals of Behavioral Medicine, 55(11),* 1062-1079.

Koo, C., Park, J., Ryu, J., & Han, S. (2020). The Effect of Virtual Reality on Preoperative Anxiety: A Meta-Analysis of Randomized Controlled Trials*. Journal of Clinical Medicine, 9(10),* 3151.

Lahti, S., Suominen, A., Freeman, R., Lähteenoja, T., & Humphris, G. (2020). Virtual reality relaxation to decrease dental anxiety: Immediate effect randomized clinical trial. *JDR Clinical & Translational Research, 5(4),* 312-318.

Lan, X., Tan, Z., Zhou, T., Huang, Z., Huang, Z., Wang, C., Chen, Z., Ma, Y., Kang, T., Gu, Y., Wang, D., & Huang, Y. (2023). Use of Virtual Reality in Burn Rehabilitation: A Systematic Review and Meta-analysis. *Archives of Physical Medicine and Rehabilitation, 104(3),* 502-513.

Li, Z., Liu, L., & Yin, Y. (2020). Effectiveness of VR glasses in primiparous delivery. *China Modern Med, 27(15),* 246-249.

Litwin, S. P., Nguyen, C., Hundert, A., Stuart, S., Liu, D., Maguire, B., Matava, C., & Stinson, J. (2021). Virtual reality to reduce procedural pain during IV insertion in the pediatric emergency department: a pilot randomized controlled trial. *The Clinical Journal of Pain, 37(2),* 94-101.

Liu, K. Y., Ninan, S. J., Laitman, B. M., Goldrich, D. Y., Iloreta, A. M., & Londino III, A. V. (2020). Virtual reality as distraction analgesia and anxiolysis for pediatric otolaryngology procedures. *The Laryngoscope, 131(5),* e1714-e1721.

Liu, X., & Wan, L. (2020). Effectiveness of virtual reality technology in reducing pain and anxiety during natural childbirth in primiparous women. *Chinese General Practice Nursing, 18(21),* 2668-2670.

López-Valverde, N., Muriel Fernández, J., López-Valverde, A., Valero Juan, L. F., Ramírez, J. M., Flores Fraile, J., Herrero Payo, J., Blanco Antona, L. A., Macedo de Sousa, B., & Bravo, M. (2020). Use of Virtual Reality for the Management of Anxiety and Pain in Dental Treatments: Systematic Review and Meta-Analysis*. Journal of Clinical Medicine, 9(4),* 1025.

Maciołek, J., Wąsek, W., Kamiński, B., Piotrowicz, K., & Krzesiński, P. (2020). The impact of mobile virtual reality–enhanced relaxation training on anxiety levels in patients undergoing cardiac rehabilitation*. Kardiologia Polska, 78(10),* 1032-1034.

McSherry, T., Atterbury, M., Gartner, S., Helmold, E., Searles, D. M., & Schulman, C. (2018). Randomized, crossover study of immersive virtual reality to decrease opioid use during painful wound care procedures in adults. *Journal of Burn Care & Research, 39(2),* 278-285.

Mitrakul, K., Asvanund, Y., Arunakul, M., & Paka-Akekaphat, S. (2015). Effect of audiovisual eyeglasses during dental treatment in 5-8 year-old children Introduction. *European Journal of Paediatric Dentistry, 16(239),* 26418930.

Mohammad, E. B., & Ahmad, M. (2019). Virtual reality as a distraction technique for pain and anxiety among patients with breast cancer: A randomized control trial. *Palliative & Supportive Care, 17(1),* 29-34.

Momenyan, N., Safaei, A. A., & Hantoushzadeh, S. (2021). Immersive virtual reality analgesia in un-medicated laboring women (during stage 1 and 2): a randomized controlled trial. *Clinical and Experimental Obstetrics & Gynecology, 48(1),* 110-116.

Morgan, H., Nana, M., Phillips, D., & Gallagher, S. (2021). The Effect of a VIrtual RealiTy Immersive Experience Upon Anxiety Levels, Procedural Understanding, and Satisfaction in Patients Undergoing CArdiac CaTHeterization: The VIRTUAL CATH Trial. *The Journal of Invasive Cardiology, 33(9),* e681-e686.

Morris, L. D., Louw, Q. A., & Crous, L. C. (2010). Feasibility and potential effect of a low-cost virtual reality system on reducing pain and anxiety in adult burn injury patients during physiotherapy in a developing country. *Burns, 36(5),* 659-664.

Niharika, P., Reddy, N. V., Srujana, P., Srikanth, K., Daneswari, V., & Geetha, K. S. (2018). Effects of distraction using virtual reality technology on pain perception and anxiety levels in children during pulp therapy of primary molars. *Journal of Indian Society of Pedodontics and Preventive Dentistry, 36(4),* 364-369.

Noben, L., Goossens, S. M. T. A., Truijens, S. E. M., Van Berckel, M. M. G., Perquin, C. W., Slooter, G. D., & Van Rooijen, S. J. (2019). A virtual reality video to improve information provision and reduce anxiety before cesarean delivery: randomized controlled trial. *JMIR mental health, 6(12),* e15872.

Nunna, M., Dasaraju, R. K., Kamatham, R., Mallineni, S. K., & Nuvvula, S. (2019). Comparative evaluation of virtual reality distraction and counter-stimulation on dental anxiety and pain perception in children. *Journal of Dental Anesthesia and Pain Medicine, 19(5),* 277.

Obrero-Gaitán, E., Cortés-Pérez, I., Calet-Fernández, T., García-López, H., López Ruiz, M. d. C., & Osuna-Pérez, M. C. (2022). Digital and Interactive Health Interventions Minimize the Physical and Psychological Impact of Breast Cancer, Increasing Women’s Quality of Life: A Systematic Review and Meta-Analysis. *Cancers, 14(17),* 4133.

Özkan, T. K., & Polat, F. (2020). The effect of virtual reality and kaleidoscope on pain and anxiety levels during venipuncture in children. Journal of *PeriAnesthesia Nursing, 35(2),* 206-211.

Pande, P., Rana, V., Srivastava, N., & Kaushik, N. (2020). Effectiveness of different behavior guidance techniques in managing children with negative behavior in a dental setting: A randomized control study. *Journal of Indian Society of Pedodontics and Preventive Dentistry, 38(3),* 259-265.

Park, J., Nahm, F., Kim, J., Jeon, Y., Ryu, J., & Han, S. (2019). The effect of mirroring display of virtual reality tour of the operating theatre on preoperative anxiety: a randomized controlled trial. *IEEE Journal of Biomedical and Health Informatics, 23(6),* 2655-2660.

Piskorz, J., & Czub, M. (2018). Effectiveness of a virtual reality intervention to minimize pediatric stress and pain intensity during venipuncture. *Journal for Specialists in Pediatric Nursing, 23(1),* e12201.

Ran, L., Zhao, N., Fan, L., Zhou, P., Zhang, C., & Yu, C. (2021). Application of virtual reality on non-drug behavioral management of short-term dental procedure in children. *Trials, 22,* 1-9.

Robertson, A., Khan, R., Fick, D., Robertson, W.B., Gunaratne, D.R., Yapa, S., Bowden, V., Hoffman, H. and Rajan, R., 2017, June. The effect of Virtual Reality in reducing preoperative anxiety in patients prior to arthroscopic knee surgery: A randomised controlled trial. In *5th IEEE International Conference on Serious Games and Applications for Health*, SeGAH 2017. IEEE, Institute of Electrical and Electronics Engineers.

Ryu, J., Park, J., Nahm, F., Jeon, Y., Oh, A., Lee, H., Kim, J., & Han, S. (2018). The effect of gamification through a virtual reality on preoperative anxiety in pediatric patients undergoing general anesthesia: a prospective, randomized, and controlled trial. *Journal of Clinical Medicine, 7(9),* 284.

Ryu, J., Park, S., Park, J., Kim, J., Yoo, H., Kim, T., Hong, J., & Han, S. (2017). Randomized clinical trial of immersive virtual reality tour of the operating theatre in children before anaesthesia. *Journal of British Surgery, 104(12),* 1628-1633.

Ryu, J., Oh, A., Yoo, H., Kim, J., Park, J., & Han, S. (2019). The effect of an immersive virtual reality tour of the operating theater on emergence delirium in children undergoing general anesthesia: A randomized controlled trial. *Pediatric Anesthesia, 29(1),* 98-105.

Schneider, S. M., & Workman, M. (1999). Effects of virtual reality on symptom distress in children receiving chemotherapy. *CyberPsychology & Behavior, 2(2),* 125-134.

Sharifpour, S., Manshaee, G. R., & Sajjadian, I. (2021). Effects of virtual reality therapy on perceived pain intensity, anxiety, catastrophising and self‐efficacy among adolescents with cancer. *Counselling and Psychotherapy Research, 21(1),* 218-226.

Shetty, V., Suresh, L. R., & Hegde, A. M. (2019). Effect of virtual reality distraction on pain and anxiety during dental treatment in 5 to 8 year old children. *Journal of Clinical Pediatric Dentistry, 43(2),* 97-102.

Simonetti, V., Tomietto, M., Comparcini, D., Vankova, N., Marcelli, S., & Cicolini, G. (2022). Effectiveness of virtual reality in the management of paediatric anxiety during the peri‑operative period: A systematic review and meta-analysis. *International Journal of Nursing Studies, 125,* 104115.

Szczepańska-Gieracha, J., Jóźwik, S., Cieślik, B., Mazurek, J., & Gajda, R. (2021). Immersive virtual reality therapy as a support for cardiac rehabilitation: a pilot randomized-controlled trial. *Cyberpsychology, Behavior, and Social Networking, 24(8),* 543-549.

Tas, F. Q., van Eijk, C. A. M., Staals, L. M., Legerstee, J. S., & Dierckx, B. (2022). Virtual reality in pediatrics, effects on pain and anxiety: A systematic review and meta-analysis update. *Pediatric Anesthesia, 32(12),* 1292-1304.

Tejera, M. D., Beltran-Alacreu, H., Cano-de-la-Cuerda, R., Leon Hernandez, J. V., Martín-Pintado-Zugasti, A., Calvo-Lobo, C., Gil-Martínez, A., & Fernández-Carnero, J. (2020). Effects of virtual reality versus exercise on pain, functional, somatosensory and psychosocial outcomes in patients with non-specific chronic neck pain: a randomized clinical trial*. International Journal of Environmental Research and Public Health, 17(16),* 5950.

Tennant, M., Youssef, G. J., McGillivray, J., Clark, T-J., McMillan, L., & McCarthy, M. C. (2020). Exploring the use of immersive virtual reality to enhance psychological well-being in pediatric oncology: a pilot randomized controlled trial. *European Journal of Oncology Nursing, 48,* 101804.

Turan Kavradim, S., Yangöz, Ş. T., & Özer, Z. (2023). Effectiveness of virtual reality interventions on physiological and psychological outcomes of adults with cardiovascular disease: A systematic review and meta-analysis. *Journal of Nursing Scholarship, 55(5),* 949-966.

Turrado, V., Guzmán, Y., Jiménez-Lillo, J., Villegas, E., de Lacy, F. B., Blanch, J., Balibrea, J. M., & Lacy, A. (2021). Exposure to virtual reality as a tool to reduce peri-operative anxiety in patients undergoing colorectal cancer surgery: a single-center prospective randomized clinical trial. *Surgical endoscopy, 35,* 4042-4047.

van Twillert, B., Bremer, M., & Faber, A. W. (2007). Computer-generated virtual reality to control pain and anxiety in pediatric and adult burn patients during wound dressing changes. *Journal of Burn Care & Research, 28(5),* 694-702.

Wang, Y., Guo, L., & Xiong, X. (2022). Effects of Virtual Reality-Based Distraction of Pain, Fear, and Anxiety During Needle-Related Procedures in Children and Adolescents. *Frontiers in Psychology,* 13.

Wolitzky, K., Fivush, R., Zimand, E., Hodges, L., & Rothbaum, B. O. (2005). Effectiveness of virtual reality distraction during a painful medical procedure in pediatric oncology patients. *Psychology and Health, 20(6),* 817-824.

Wong, C. L., Li, C. K., Chan, C. W., Choi, K. C., Chen, J., Yeung, M. T., & Chan, O. N. (2020). Virtual reality intervention targeting pain and anxiety among pediatric cancer patients undergoing peripheral intravenous cannulation: a randomized controlled trial. *Cancer Nursing, 44(6),* 435-442.

Wu, Y., Wang, N., Zhang, H., Sun, X., Wang, Y., & Zhang, Y. (2023). Effectiveness of Virtual Reality in Symptom Management of Cancer Patients: A Systematic Review and Meta-Analysis. *Journal of Pain and Symptom Management, 65(5),* e467-e482.

Xu, N., Chen, S., Liu, Y., Jing, Y., & Gu, P. (2022). The Effects of Virtual Reality in Maternal Delivery: Systematic Review and Meta-analysis. *JMIR Serious Games, 10(4),* e36695.

Yan, X., Yan, Y., Cao, M., Xie, W., O'Connor, S., Lee, J. J., & Ho, M-H. (2023). Effectiveness of virtual reality distraction interventions to reduce dental anxiety in paediatric patients: A systematic review and meta-analysis. Journal of Dentistry, 132, 104455.

Yang, J., Ryu, J., Nam, E., Lee, H., & Lee, J. (2019). Effects of preoperative virtual reality magnetic resonance imaging on preoperative anxiety in patients undergoing arthroscopic knee surgery: a randomized controlled study. *Arthroscopy: The Journal of Arthroscopic & Related Surgery, 35(8),* 2394-2399.

Zeng, Y., Zhang, J-E., Cheng, A., Cheng, H., & Wefel, J. (2019). Meta-Analysis of the Efficacy of Virtual Reality–Based Interventions in Cancer-Related Symptom Management. Integrative Cancer Therapies, 18, 1534735419871108.

Zhang, H., Xu, H., Zhang, Z-X., & Zhang, Q. (2022). Efficacy of virtual reality-based interventions for patients with breast cancer symptom and rehabilitation management: a systematic review and meta-analysis. *BMJ Open, 12(3),* e051808.
