# Supplementary material for: Extended Reality Interventions for Health and Procedural Anxiety: Panoramic Meta-Analysis Based on Overviews of Reviews
Source: J Med Internet Res. 2025 Jan 8;27:e58086. doi: 10.2196/58086 (PMC11754977; doi:10.2196/58086)
Supplement: Multimedia Appendix 5 [file jmir_v27i1e58086_app5.docx]

**Multimedia Appendix 5.** Funnel plots for publication bias
